# Discovery of Bispecific Lead Compounds from *Azadirachta indica* against ZIKA NS2B-NS3 Protease and NS5 RNA Dependent RNA Polymerase Using Molecular Simulations

**DOI:** 10.3390/molecules27082562

**Published:** 2022-04-15

**Authors:** Sanjay Kumar, Sherif A. El-Kafrawy, Shiv Bharadwaj, S. S. Maitra, Thamir A. Alandijany, Arwa A. Faizo, Aiah M. Khateb, Vivek Dhar Dwivedi, Esam I. Azhar

**Affiliations:** 1 School of Biotechnology, Jawaharlal Nehru University, New Delhi 110067, India; sanjay51_sbt@jnu.ac.in (S.K.); ssm2100@mail.jnu.ac.in (S.S.M.); 2 Center for Bioinformatics, Computational and Systems Biology, Pathfinder Research and Training Foundation, Greater Noida 201308, India; 3 Special Infectious Agents Unit, King Fahd Medical Research Center, King Abdulaziz University, P.O. Box 128442, Jeddah 21362, Saudi Arabia; saelkfrawy@kau.edu.sa (S.A.E.-K.); talandijany@kau.edu.sa (T.A.A.); aafaizo@kau.edu.sa (A.A.F.); akhateb@taibahu.edu.sa (A.M.K.); 4 Department of Medical Laboratory Sciences, Faculty of Applied Medical Science, King Abdulaziz University, Jeddah 21589, Saudi Arabia; 5 Laboratory of Ligand Engineering, Institute of Biotechnology of the Czech Academy of Sciences v.v.i., BIOCEV Research Center, 252 50 Vestec, Czech Republic; 6 Medical Laboratory Technology Department, College of Applied Medical Sciences, Taibah University, Medina 42353, Saudi Arabia; 7 Institute of Advanced Materials, IAAM, 59053 Ulrika, Sweden

**Keywords:** Zika virus, NS2B-NS3 protease, NS5 RdRp, therapeutics, molecular dynamics, flavonoids, *Azadirachta indica*

## Abstract

Zika virus (ZIKV) has been characterized as one of many potential pathogens and placed under future epidemic outbreaks by the WHO. However, a lack of potential therapeutics can result in an uncontrolled pandemic as with other human pandemic viruses. Therefore, prioritized effective therapeutics development has been recommended against ZIKV. In this context, the present study adopted a strategy to explore the lead compounds from *Azadirachta indica* against ZIKV via concurrent inhibition of the NS2B-NS3 protease (ZIKV^pro^) and NS5 RNA dependent RNA polymerase (ZIKV^RdRp^) proteins using molecular simulations. Initially, structure-based virtual screening of 44 bioflavonoids reported in *Azadirachta indica* against the crystal structures of targeted ZIKV proteins resulted in the identification of the top four common bioflavonoids, viz. Rutin, Nicotiflorin, Isoquercitrin, and Hyperoside. These compounds showed substantial docking energy (−7.9 to −11.01 kcal/mol) and intermolecular interactions with essential residues of ZIKV^pro^ (B:His^51^, B:Asp^75^, and B:Ser^135^) and ZIKV^RdRp^ (Asp^540^, Ile^799^, and Asp^665^) by comparison to the reference compounds, O7N inhibitor (ZIKV^pro^) and Sofosbuvir inhibitor (ZIKV^RdRp^). Besides, long interval molecular dynamics simulation (500 ns) on the selected docked poses reveals stability of the respective docked poses contributed by intermolecular hydrogen bonds and hydrophobic interactions. The predicted complex stability was further supported by calculated end-point binding free energy using molecular mechanics generalized born surface area (MM/GBSA) method. Consequently, the identified common bioflavonoids are recommended as promising therapeutic inhibitors of ZIKV^pro^ and ZIKV^RdRp^ against ZIKV for further experimental assessment.

## 1. Introduction

Zika virus (ZIKV) was first isolated in 1947 from Zika forest, Uganda, East Africa [1], and remained unnoticed for almost 60 years. In 2007, this virus caught everyone’s attention following the first-ever ZIKV epidemic outbreak in Yap Island, Federated States of Micronesia, where 59 predictable and 49 confirmed ZIKV cases were reported [2]. Since then, ZIKV has caused several epidemics outside African countries in the last ten years, including the 2013–2014 outbreak in French Polynesia, infecting around 28,000 people [3,4]. Subsequently, in 2015, suspected outbreak of ZIKV in Brazil was estimated to infect 440,000 to 1,300,000 individuals [5], while microcephaly and other neurological disorders were also observed in approximately 7000 infected individuals [6,7]. In 2016, several cases of ZIKV infection were observed in females from the United States of America (USA) who had never travelled to the countries affected with this virus, but their male partners did [8]; the presence of ZIKV in their semen confirmed that it could also be transmitted through sexual contact [9]. Notably, similar to all of the flaviviruses, ZIKV is also primarily transmitted through *Aedes aegypti* mosquitoes [10]. However, the transfer of ZIKA infection through sexual transmission [8] and the vertical transmission from mother to the fetus [11,12,13] marks this virus as a global health concern. Currently, no therapeutics or treatments are available for the ZIKV infection. As a consequence, ZIKA is posing a serious threat to humans globally. Thus, this raises a demand for the development of potential therapeutics to control the ZIKA epidemic and associated neurological disorders.

**Figure 1:**
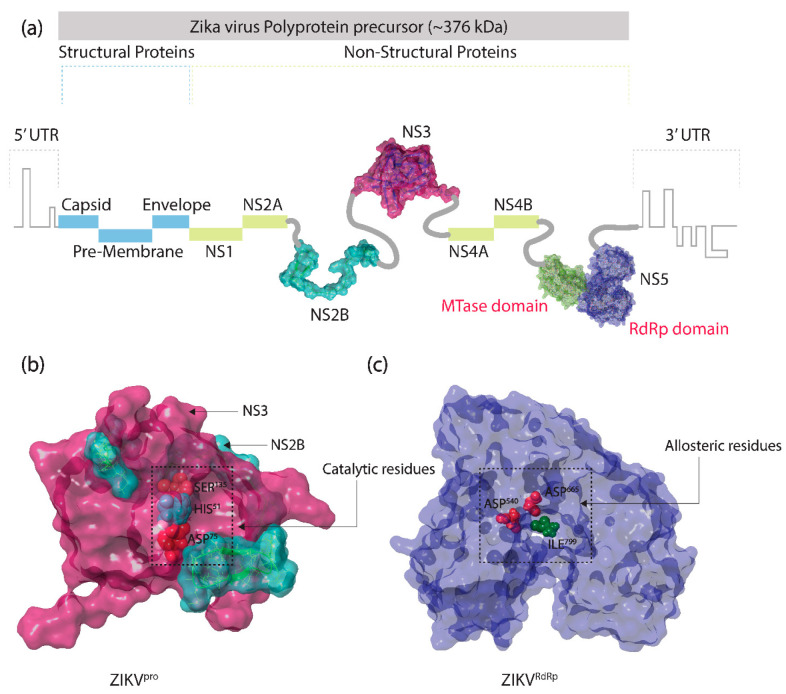
Zika virus (ZIKV) polyprotein structure: (**a**) arrangement of structural and non-structural proteins in a single polyprotein encoded by the ~10.8 kb RNA genome of ZIKV, (**b**) three-dimensional (3D) crystal structures of ZIKV^pro^ of resolution 1.59 Å retrieved from the Protein Data Bank (PDB) with PDB ID: 6Y3B, and (**c**) 3D crystal structures of ZIKV^RdRp^ of resolution 1.40 Å retrieved from PDB with PDB ID: 6LD2. All of the 3D structures of proteins were prepared and modified using free Maestro academic version v12.9 package (Schrödinger Release 2021-3: Maestro, Schrödinger, LLC, New York, NY, USA, 2021).

ZIKV is a vector-borne envelop flavivirus and encloses a 10.8 kb positive sense, single-stranded, RNA (+ssRNA) genome, which contains a single open reading frame (ORF) for the translation of a single polyprotein of 3419 amino acids [14]. Genome replication plays a central role in the viral pathogenesis. Thus, after infection, ZIKV polyprotein is processed into three structural proteins (SPs): pre-membrane (prM), envelope (E), and capsid (C) proteins, and seven nonstructural proteins (NSPs): NS1, NS2A, NS2B, NS3, NS4A, NS4B, and NS5, via the proteolytic activity of both ZIKA and host proteinases (Figure 1) [15]. The structural proteins provide the protection to the newly synthesized viral genome by forming an inner layer of capsid proteins while the precursor membrane (prM) protein and an envelope (E) protein contribute to the formation of the virion surface. During maturation, the prM protein is proteolytically cleaved into pr-subunit and M-subunit by the catalytic activity of host furin protease in the trans-Golgi network (TGN). This event results in the formation and release of fully matured ZIKV with E and M protein on its outer envelope from the host cell [16]. Genome replication is the crux of viral pathogenesis, and in the case of ZIKV, the NSPs interact to form a replication complex that provides a site for the synthesis of RNA genome of the viral genomic RNA. Among all of the NSPs, NS2B-NS3 protease (ZIKV^pro^) and NS5 RNA dependent RNA polymerase (ZIKV^RdRp^) are the vital factors in ZIKV pathogenesis, as the former one is involved in the hydrolysis and maturation of the flavivirus polyprotein whereas the latter one has polymerase activity, which is necessary for the viral replication process [17].

The ZIKV serine protease (ZIKV^pro^), a heterodimeric complex, is consists of a membrane protein NS2B bound with ~70 kDa NS3 protein at the N-terminal region [18,19,20]. The NS3 protein has protease and helicase domains at the N-terminal and the C-terminal, respectively. However, despite lacking any enzymatic activity, NS2B plays a crucial role in the folding of NS3 protein [21,22,23]—acted as a co-factor for the activity of NS3 protein [24] and holds it near the cell membrane, which is essential for its proteolytic activity and viral replication [25,26,27,28,29]. Thus, in NS2B-NS3 protease (ZIKV^pro^), the substrate binding and catalyzing active site of the NS3 protease domain is enfolded by the NS2B protein. Herein, a stretch of forty amino acid residues located at the C-terminal region of NS2B interact with the N-terminal protease domain of NS3 protein [30,31,32,33,34,35], which results in the formation of a catalytic triad (His^51^, Asp^75^, and Ser^135^ residues).

These catalytic residues are required for the proteolytic activity by the virus to release the functional NSPs in the cytosolic side of the host endoplasmic reticulum (ER), which further participate in viral replication (Figure 1) [36,37]. Due to this crucial role of ZIKV^pro^ in the life cycle ZIKV and the lack of any a homolog in human cells, ZIKV^pro^ is considered as a promising target for anti-ZIKV drug development.

Moreover, NS5 is the largest and highly conserved protein among flaviviruses, including ZIKV, which showed essential function in the viral genome replication via the C-terminal NS5 RdRp (ZIKV^RdRp^) domain. Therefore, targeting the ZIKV^RdRp^ domain has been considered as a precise therapeutic strategy against ZIKV [38,39,40]. The structural analysis revealed the right-hand-shaped conformation for the ZIKV^RdRp^ domain, holding three main domains: fingers, palm, and thumb, where the finger and thumb domains intersect to form an active region with a central catalytic pocket formed by the palm domain. Herein, the amino acids ranging from 321 to 488 and 542 to 608 comprise the finger domain, 484 to 541 and 609 to 714 comprise the palm domain, and 715 to 903 comprise the thumb domain, where Asp^540^ (palm domain) and Ile^799^ and Asp^665^ residues (thumb domain) were identified to form the catalytic site of ZIKV^RdRp^ and crucial for the interaction with the ligands (Figure 1) [41,42]. In addition, NS5 protein also carries methyltransferase (MTase) activity at the N-terminal end, which is required for the 5′ capping of newly synthesized viral mRNA [43].

In 2016, after the WHO announced the ZIKV outbreak with a global health emergency, various therapeutics, such as orthosteric inhibitors [44,45], allosteric inhibitors [46,47,48], ZIKV^pro^ inhibitors [49,50], ZIKV^RdRp^ inhibitors [51,52,53,54], and a few inhibitors with unknown molecular targets [55,56,57], were reported as a treatment for the ZIKV infection. However, only one compound, viz. novobiocin, was noted for considerable in vivo inhibitory effect against the ZIKV infection [58]. Therefore, in the war against ZIKV, a comprehensive blueprint is needed to develop a promising anti-ZIKV therapeutics. In this context, among the various therapeutic designing methods, a multitargeted approach has been suggested as an aspiring strategy where the most appealing targets for the ZIKV are ZIKV^pro^ and ZIKV^RdRp^ domains.

In the last two decades, the development of multitargeted drugs has been taken into preference due to their major advantages, due to the lower risk for drug interactions and improved drug compliance in patients [59,60,61]. In this context, the present study opted the multitargeting approach against ZIKV by identification of potent bioflavonoids as ZIKV^pro^ and ZIKV^RdRp^ domain putative inhibitors from the *Azadirachta indica,* popularly known as Neem, which is well established to possess antibacterial, antifungal, and antiviral activity [62]. Of note, the various parts of the *A. indica,* such as leaves, flowers, bark, seeds, and roots, are used in several therapies and treatments for the infectious and non-infectious diseases in Asian and African countries since time immemorial. In recent years, medicinal plants, including *A. indica,* are the foremost choice in finding a cure against numerous diseases due to their least toxicity, unique chemistry of secondary metabolites, and a long-term resource with constant mass production [63,64]. Therefore, in this study, 44 bioflavonoids reported from *Azadirachta indica,* were computationally screened in the active pocket of ZIKV^pro^ and ZIKV^RdRp^ domains to identify bispecific potent inhibitors with substantial binding affinity and stability for the drug development against ZIKV infection.

## 2. Computational Methods

### 2.1. Receptors and Bioflavonoids

The three-dimensional (3D) crystal structure of ZIKV NS2B-NS3 protease (ZIKV^pro^, PDB ID: 6Y3B [65]) and ZIKV NS5 RNA-dependent RNA polymerase domain (ZIKV^RdRp^, PDB ID: 6LD2 [52]) of 1.59 and 1.40 Å resolutions, respectively were downloaded from the protein data bank (PDB) database (https://www.rcsb.org/) [66]. The selected proteins as receptors were preprocessed by assigning bond order and addition of polar hydrogen atoms using the default parameters in Protein preparation wizard of the Maestro-Schrödinger suite [67]. Herein, protein structures were treated for protonation of residues using the PROPKA program at pH 7.0, followed by the restrained minimization using Optimized Potentials for Liquid Simulations 3e (OPLS3e) force field under default parameters.

To identify the bioflavonoids from *Azadirachta indica* (Neem plant) as putative inhibitors of ZIKV^pro^ and ZIKV^RdRp^, a small library of known 44 bioflavonoids was prepared by exploring the documented research articles (Table S1). The three-dimensional conformers of all bioflavonoids were retrieved from the PubChem database (https://pubchem.ncbi.nlm.nih.gov/) [68] and treated as ligand for the computational analysis against targeted ZIKV proteins. Briefly, the ligand library was prepared under default parameters using LigPrep panel in Schrödinger suite (Schrödinger Release 2018-3: LigPrep, Schrödinger, LLC, New York, NY, USA, 2018), where ligand tautomeric conformations were generated using EPIK state penalty at pH 7.0 ± 2.0 with OPLS3e force field.

### 2.2. Structure-Based Virtual Screening and ADMET Analysis

In the initial stage of drug discovery, structure-based virtual screening (SBVS) plays a crucial role in identifying the novel bioactive molecules as potent ligand against the three-dimensional structure of a certain biological targets obtained through X-ray diffraction, NMR, Cryo-electron microscopy, or homology model. SBVS is a computational technique which attempts to predict the putative conformations between the receptor and ligand for complex formation and uses the non-covalent interactions-based scoring function to mark the stability of calculated receptor-ligand complexes.

In search of bispecific putative inhibitors from *Azadirachta indica* for ZIKV treatment*,* a total of 44 bioflavonoids were considered for SBVS against the active pocket of ZIKV^pro^ and ZIKV^RdRp^ using Glide extra precision (XP) module of Schrödinger suite (Schrödinger Release 2018-3: Glide, Schrödinger, LLC, New York, NY, USA, 2018). Herein, the docking grid was prepared around the catalytic site residues of ZIKV^pro^ (B:His^51^, B:Asp^75^, and B:Ser^135^ residues) [66] and allosteric pocket of ZIKV^RdRp^ (Asn^612^, Asp^665^, Asp^666^, Cys^711^, Thr^796^, Try^797^, Ser^798^, Ile^799^, and His^800^ residues) under default parameters using Grid generation tool of Schrödinger suite [52,69]. Following, based on the docking XP score, the top common bioflavonoids with significant binding energy were extracted as putative inhibitors of the selected proteins of ZIKV. Furthermore, identified compounds were also computed for their pharmacokinetic/drug-like properties via ADMET analysis using SwissADME (http://www.swissadme.ch/) [70] and admetSAR (http://lmmd.ecust.edu.cn/admetsar2/) [71] online servers.

### 2.3. Redocking and Intermolecular Interaction Profiling

The top common compounds collected from SBVS against ZIKV proteins, i.e., ZIKV^pro^ and ZIKV^RdRp^, and respective reference compounds, i.e., O7N inhibitor (native co-crystalized ligand) for ZIKV^pro^ and Sofosbuvir inhibitor for ZIKV^RdRp^ (previously reported nucleoside inhibitor of ZIKV^RdRp^) [72], were redocked in the selected respective binding pockets of viral proteins under default parameters using Glide XP module of Schrödinger suite (Schrödinger Release 2018-3: Glide, Schrödinger, LLC, New York, NY, USA, 2018). All of the docked poses were studied for the intermolecular interactions under the default parameters in the Maestro v12.9 package, and both 3D and 2D interaction diagrams were prepared using the free academic version of the Maestro v12.9 package (Schrödinger Release 2021-3: Maestro, Schrödinger, LLC, New York, NY, USA, 2021).

### 2.4. Molecular Dynamics Simulation Analysis

Dynamic stability and the intermolecular interactions profiling of the selected protein-ligand complexes were analyzed through the molecular dynamics (MD) simulation using free academic Desmond-maestro 2020-4 package [73,74]. Initially, each docked complex was placed in a 10 Å × 10 Å × 10 Å orthorhombic box amended with explicit (TIP4P: transferable intermolecular potential 4 point) solvent using a system builder module. Following, the complete simulation system was amended with 0.15 M salt to mimic the physiological conditions and neutralized using counter sodium and chlorine ions while placed at 20 Å distance from the docked ligand in the binding pocket of the receptor. Later, the simulation system was minimized under default parameters using a minimization tool and subjected to 500 ns simulation under OPLS-2005 force field at 300 K temperature and 1.01325 bar pressure with default parameters using Molecular dynamics simulation tool of free academic Desmond-maestro 2020-4 [73,74]. At last, the MD simulation trajectory of each protein-ligand complex was analyzed for the stability and intermolecular interactions as a function of 500 ns interval by simulation interaction diagram (SID) module in the free academic Desmond-maestro 2020-4 suite [73,74].

The following Equation (1) calculated the root mean square deviation (RMSD) values for the protein alpha carbon (Cα) atoms and the ligand heavy atoms with respect to protein (Cα) in each frame during the 500 ns simulation trajectory to measure the average deviation that occurred in the protein and ligand for the respective docked complex in reference to their respective initial poses [75].

(1) $${\mathrm{RMSD}}_{x}=\sqrt{\frac{1}{N}}\overset{N}{\underset{i=1}{\sum }}{\left(\;{r^{\prime }}_{i}\;\left(t_{x}\right)-r_{i}\left(t_{ref}\right)\right)}^{2}$$

While calculating RMSD values, *N* represents the number of atoms selected; *t_ref_* is defined as reference time at zero interval; *r_i_* denotes the position of the atoms under evaluation in frame *x* followed by the superimposition on the reference frame *r′_i_* at time interval *t_x_*.

Moreover, root mean square fluctuation (RMSF) values were also calculated for characterizing the local fluctuations at residue and atomic level in protein structure and ligand molecule, respectively. The following equation (2) expresses the local fluctuation in the simulation trajectory [75].

(2) $${\mathrm{RMSF}}_{i}=\sqrt{\frac{1}{T}}\overset{T}{\underset{t=1}{\sum }}{\left(\;{r^{\prime }}_{i}\;\left(t\right)-r_{i}\left(t_{ref}\right)\right)}^{2}$$

While calculating RMSF, *T* denotes the simulation interval for which the RMSF is calculated, *t_ref_* denotes the reference time, *r_i_* denotes the atom position in reference time *t_ref_* and *r′_i_* denotes atom position at the time following superimposition on the reference frame.

### 2.5. Endpoint Free Binding Energy Calculation

Molecular mechanics/generalized Born Surface area (MM/GBSA) analysis was performed to calculate the mean binding free energy on the extracted poses from the last 10 ns interval (at 10 ps step) of respective MD simulation trajectory under default parameters with OPLS-2005 force field in the Prime MMGBSA module in Schrödinger suite (Schrödinger Release 2018-3: Prime, Schrödinger, LLC, New York, NY, USA, 2018). Herein, all of the solvent molecules and ions were deleted from the extracted poses, and the binding free energy (*Δ*G) was calculated using the following Equation (3).

(3) $$\;\Delta G_{Bind}=\Delta G_{Complex\;\left(minimized\right)}-\left(\Delta {G\;}_{Complex\;\left(minimized\right)}+\Delta {G\;}_{Ligand\;\left(minimized\right)}\right)\;$$

Where, *Δ*G*_Bind_* = Binding free energy, *Δ*G*_Complex_*_(*minimized*)_ = Free energy of the complex, *Δ*G*_Receptor_*_(*minimized*)_ = Free energy for the receptor, and *Δ*G*_Ligand_*_(*minimized*)_ = Free energy for the ligand.

## 3. Results and Discussion

### 3.1. Structure-Based Virtual Screening

**Figure 2:**
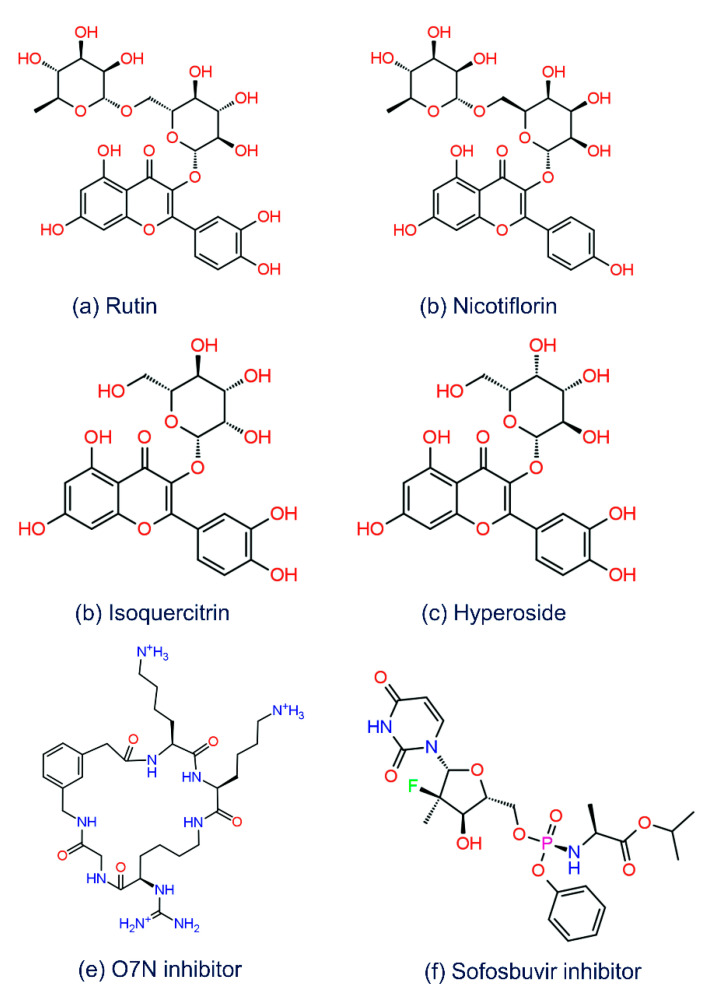
2D structures of selected bioflavonoids, i.e., (**a**) Rutin, (**b**) Nicotiflorin, (**c**) Isoquercitrin, and (**d**) Hyperoside, as well as reference compounds, i.e., (**e**) O7N inhibitor for ZIKV^pro^, and (**f**) Sofosbuvir inhibitor for ZIKV^RdRp^.

The primary goal of this study was to find the common compounds from a natural source that can inhibit both the ZIKV^pro^ and ZIKV^RdRp^ proteins for the treatment of ZIKV infection. Thus, a small library of 44 bioflavonoids (Table S1) belonging to the *A. indica* was used in SBVS against the selected binding pocket of ZIKV^pro^ and ZIKV^RdRp^. This resultant in the collection of 21 compounds with docking scores between −2.0 to −11.01 kcal/mol against the selected viral proteins (Tables S2 and S3). Following, based on their docking scores, only the top four common bioflavonoids, i.e., Rutin, Nicotiflorin, Isoquercitrin, and Hyperoside, were marked as bispecific inhibitors for further redocking and intermolecular interaction (IMI) analysis by comparison to the reference compounds of ZIKV^pro^ and ZIKV^RdRp^, i.e., O7N inhibitor and Sofosbuvir inhibitor, respectively (Figure 2). Herein, the selected four bioactive bioflavonoids showed significant docking scores between −8 to −11.1 kcal/mol with the target proteins, i.e., ZIKV^pro^ and ZIKV^RdRp^ domain (Tables S2 and S3). Interestingly, all of the identified bioflavonoids were previously reported to have medicinal and therapeutic properties; for instance, Rutin and Isoquercitrin were documented for antiviral, anticancer, and antidiabetic activities [76,77,78,79,80,81,82,83], Nicotiflorin was described to inhibit SARS-CoV-2 M^pro^ and dengue virus NS2B-NS3 protease [75,84,85], and Hyperoside was also reported to have anticancer activity [86,87].

### 3.2. Redocking and Intermolecular Interaction Analysis

Redocking is a mandatory analysis after SBVS calculation to assure that the compounds identified and selected through virtual screening have high affinity with the active site residues of the binding pocket since the algorithms of SBVS are fast and, therefore, their accuracy level is comparatively low than the docking scoring methods [88]. Thus, a stringent XP docking protocol was adopted in the redocking of the selected poses, and the most satisfactory binding poses with substantial binding scores and interactions with the essential residues in the viral proteins, i.e., ZIKV^pro^ and ZIKV^RdRp^, were extracted for further analysis. Herein, the redocked complexes of ZIKV^pro^ with Rutin, Nicotiflorin, Isoquercitrin, and Hyperoside were noted for docking energy of −10.61, −9.95, −8.63, and −8.37 kcal/mol, respectively (Table 1). Likewise, Rutin, Nicotiflorin, Isoquercitrin, and Hyperoside compounds docked with ZIKV^RdRp^ showed higher docking scores of −11.01, −10.56, −8.84, and −7.87 kcal/mol, respectively (Table 1). Interestingly, all four bioactive bioflavonoids, i.e., Rutin, Nicotiflorin, Isoquercitrin, and Hyperoside, demonstrated higher redocking scores (−7.8 to 11.01 kcal/mol) with both the viral target proteins by comparison to the respective reference inhibitors, viz. O7N inhibitor for ZIKV^pro^ (−6.629 kcal/mol) and Sofosbuvir inhibitor for ZIKV^RdRp^ (−6.033 kcal/mol). Therefore, the redocking results concluded that each of the selected conformations of the docked bioflavonoids have established a considerable binding affinity with the binding pocket of selected viral targets, i.e., ZIKV^pro^ and ZIKV^RdRp^, and can considered for computational analysis.

**Figure 3:**
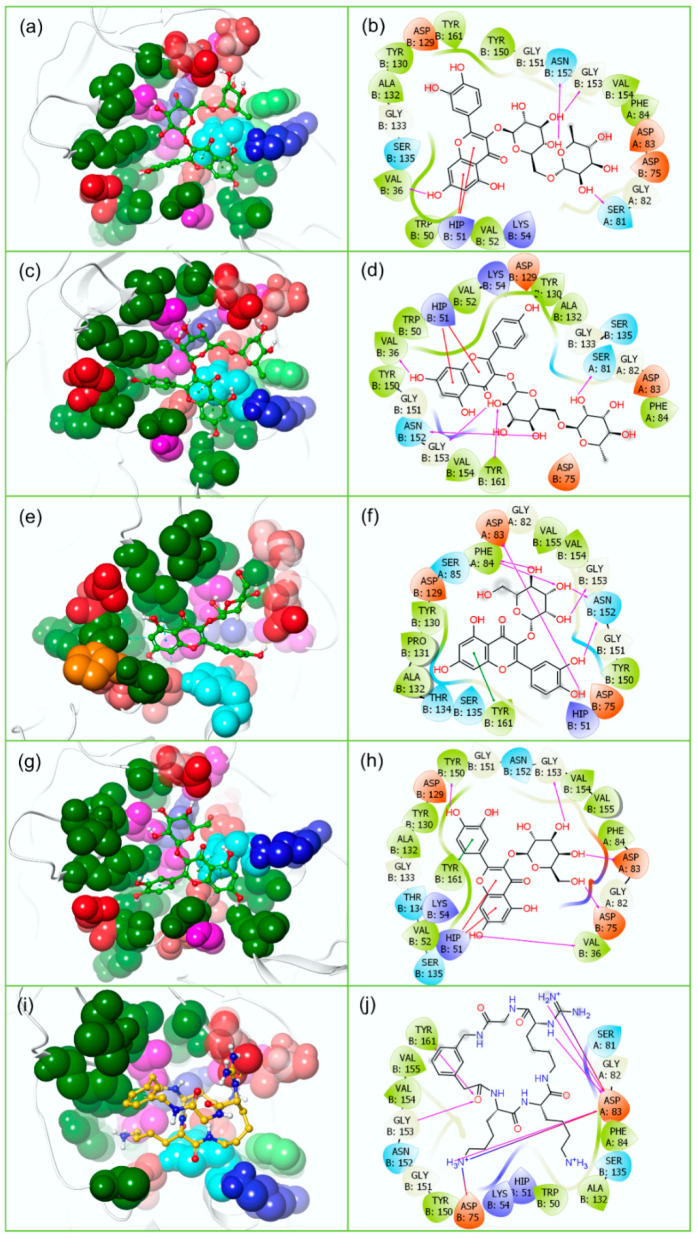
3D and 2D interaction profiles for ZIKV^pro^−bioflavonoids; (**a**,**b**) ZIKV^pro^−Rutin, (**c**,**d**) ZIKV^pro^−Nicotiflorin, (**e**,**f**) ZIKV^pro^−Isoquercitrin, (**g**,**h**) ZIKV^pro^−Hyperoside, and (**i**,**j**) ZIKV^pro^−O7N inhibitor. In 3D poses and 2D poses active residues are depicted based on residue type feature in Maestro v12.9 package, around the docked ligand at 4 Å area in the active pocket of ZIKV^pro^.

**Figure 4:**
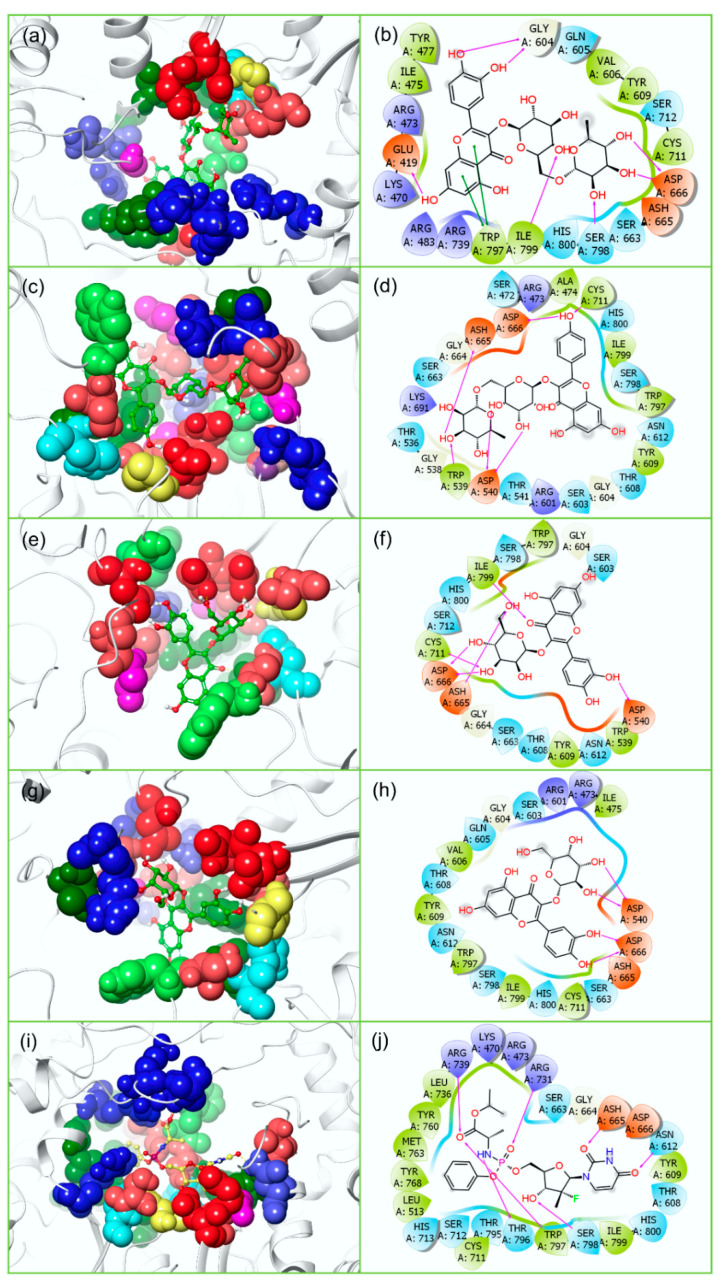
3D and 2D interaction profiles for ZIKV^RdRp^-bioflavonoids; (**a**,**b**) ZIKV^RdRp^−Rutin, (**c**,**d**) ZIKV^RdRp^−Nicotiflorin, (**e**,**f**) ZIKV^RdRp^−Isoquercitrin, and (**g**,**h**) ZIKV^RdRp^−Hyperoside, (**i**,**j**) ZIKV^RdRp^−Sofosbuvir inhibitor. In 3D poses and 2D poses, active residues are depicted based on residue type feature in the Maestro v12.9 package, around the docked ligand at 4 Å area in the active pocket of ZIKV^RdRp^.

Intermolecular interaction (IMI) analysis is essential to understand the mode of molecular contact formation between the docked ligands and the target proteins. Herein, each docked bioflavonoid (Rutin, Nicotiflorin, Isoquercitrin, and Hyperoside) was observed for the formation of hydrogen bond (H-bond), hydrophobic, and π-π interactions with the essential residues of target proteins (ZIKV^pro^ and ZIKV^RdRp^) (Table 1 and Table S4 and Figure 3 and Figure 4). In details, the docked complex of ZIKV^pro^−Rutin was observed for the formation of four H-bonds via B:Val^36^, A:Ser^81^, B:Asn^152^, and B:Gly^153^ residues, and two π-cation stacking interactions with B:His^51^ residue (Figure 3). Also, ZIKV^pro^−Nicotiflorin docked complex was noted for the formation of five H-bonds at B:Val^36^, A:Ser^81^, B:Asn^152^, B:Gly^153^, and B:Tyr^161^ residues and two π-cation stacking interactions with B:His^51^ residue (Figure 3). Likewise, ZIKV^pro^−Isoquercitrin complex exhibits formation of six H-bonds with four residues: A:Asp^83^, A:Phe^84^, B:Asn^152^, and B:Gly^153^, and one π-π stacking interactions with B:Tyr^161^ residue (Figure 3). Similarly, ZIKV^pro^−Hyperoside complex was noted for stabilization via five H-bonds formation with B:Val^36^, B:Asp^75^, A:Asp^83^, B:Tyr^150^, and B:Gly^153^ residues, two π-cation stacking interactions with B:His^51^ residue, and one π-π stacking interactions with B:Tyr^161^ residue (Figure 3). Additionally, all ZIKV^pro^−bioflavonoids docked complexes were identified for intermolecular hydrophobic, polar, negative, positive, and glycine interactions (Table 1 and Table S4, Figure 3).

Furthermore, the docked complex of ZIKV^RdRp^−Rutin was also observed for the formation of seven H-bonds with Glu^419^, Gly^604^, Asp^666^, Ser^798^, and Ile^799^ residues and two π-π stacking interactions with Trp^797^ residue (Figure 4). Whereas the ZIKV^RdRp^−Nicotiflorin docked complex exhibits the formation of six H-bonds with Asp^540^, Trp^539^, Asp^665^, Asp^666^, and Cys^711^ residues while ZIKV^RdRp^−Isoquercitrin complex showed only five H-bonds with Asp^540^, Asp^665^, Asp^666^, Cys^711^, and Ile^799^ residues (Figure 4). Compared to other complexes, ZIKV^RdRp^-Hyperoside docked complex included only two amino acid residues (Asp^540^, and Asp^666^) to form four hydrogen bonds (Figure 4). Additionally, hydrophobic, polar, positive, negative, and glycine interactions were also observed in all of the ZIKV^RdRp^−Ligand docked complexes (Figure 4, Table 1 and Table S4). Notably, a similar intermolecular interaction profile was observed in the reference docked complexes, i.e., ZIKV^pro^−O7N (Figure 3) and ZIKV^RdRp^−Sofosbuvir (Figure 4, Table 1 and Table S4). Collectively, the analysis of interaction profiles of all of the docked poses advocates the identified bispecific bioflavonoids for occupying similar active regions in targeted viral protein with higher binding energy by comparison to the respective reference inhibitors.

### 3.3. ADMET and Drug-Likeliness Analysis

In the field of drug discovery, the compounds or molecules proposed as a drug candidate must carry high biological activity and no or least toxicity. Therefore, a few critical pharmacological parameters, such as absorption, distribution, metabolism, excretion, and toxicity (ADMET parameters) along with the pharmacokinetics, has been suggested for the validation on every proposed drug candidate. Early assessments of such parameters in the initial phase of drug discovery are essential to understand and avoid drug molecules’ pharmacokinetics-related failure during clinical trials [89]. Thus, to analyze the pharmacokinetic properties and drug-likeness, all of the screened bioactive bioflavonoids, i.e., Rutin, Nicotiflorin, Isoquercitrin, and Hyperoside, as well as the reference compounds, i.e., O7N inhibitor (for ZIKV^pro^) and Sofosbuvir inhibitor (for ZIKV^RdRp^) (Figure 2), were uploaded on the SwissADME and admetSAR online servers for the assessment of ADMET properties (Tables S6 and S7). Of note, selected bioflavonoids were found to be non-inhibitor of several cytochromes (CY) such as CYP2D6, CYP1A2, CYP2C19, CYP2C9, CYP2D6, and CYP3A4, which plays a crucial role in the drug metabolism as well as various xenobiotics; an inhibition of these cytochromes may lead to the reduced drug efficacy, drug activation, and drug metabolism. Also, the selected four bioflavonoids exhibit low gastrointestinal absorption along with a lack of Blood-Brain Barrier (BBB) permeability. However, Rutin and Nicotiflorin showed three violations while Isoquercitrin and Hyperoside showed two violations for the Lipinski’s rule of (Tables S6 and S7). The selected bioflavonoids also showed violations against several other rules related to drug-likeness, such as Ghose, Veber, Egan, and Muegge. However, the rules for drug-likeness are not mandatory to be fulfilled by natural compounds as cells distinguish the bioactive compounds through the active transport system [90,91]. Additionally, several other properties related to medicinal chemistry and pharmacokinetics were evaluated for potential four compounds (Tables S6 and S7). Importantly, all of the bioflavonoids showed the negative AMES toxicity test and non-carcinogenic profiles via admetSAR server. Moreover, Conclusively, ADMET analysis suggested the selected bioflavonoids against the ZIKV^pro^ and ZIKV^RdRp^ proteins with ideal medicinal properties.

### 3.4. Long Interval Molecular Dynamics Simulation

In the field of drug discovery using computational approaches, MD simulation is an imperative technique by which the dynamic stability and the formation of intermolecular interactions of docked protein-ligand complexes are analyzed with respect to time.

**Figure 5:**
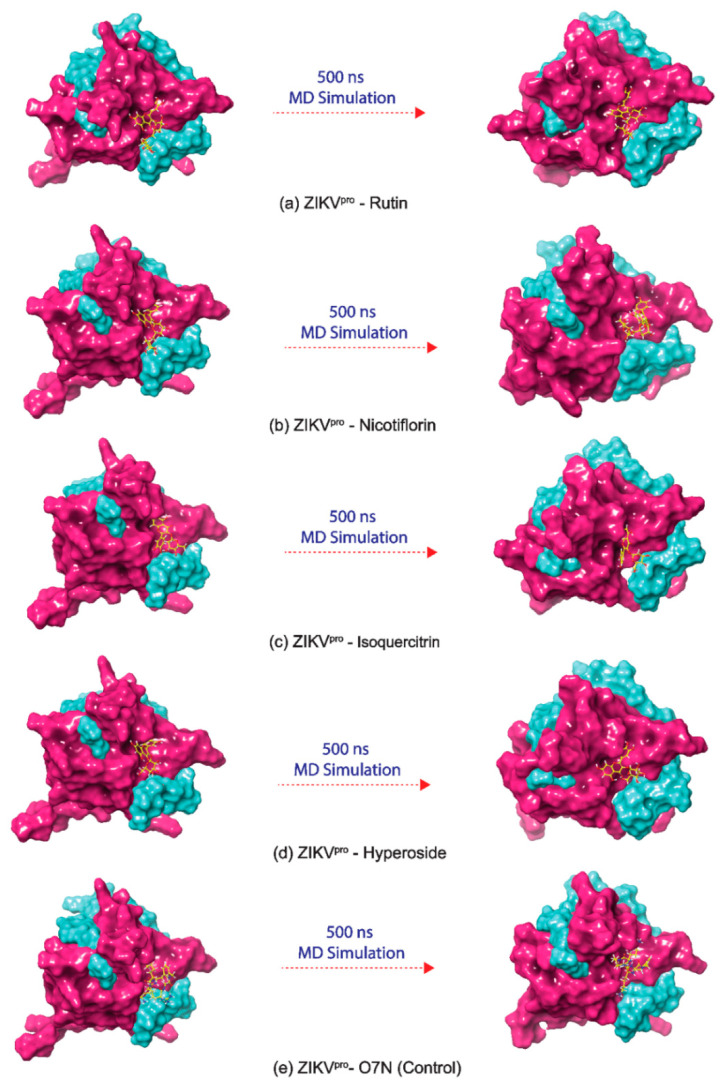
3D docked poses of ZIKV^pro^−bioflavonoids, i.e., (**a**) ZIKV^pro^−Rutin, (**b**) ZIKV^pro^−Nicotiflorin, (**c**) ZIKV^pro^−Isoquercitrin, (**d**) ZIKV^pro^−Hyperoside, and reference complex poses, i.e., (**e**) ZIKV^pro^−O7N inhibitor, exhibiting transition of docked poses through 500 ns MD simulation interval.

**Figure 6:**
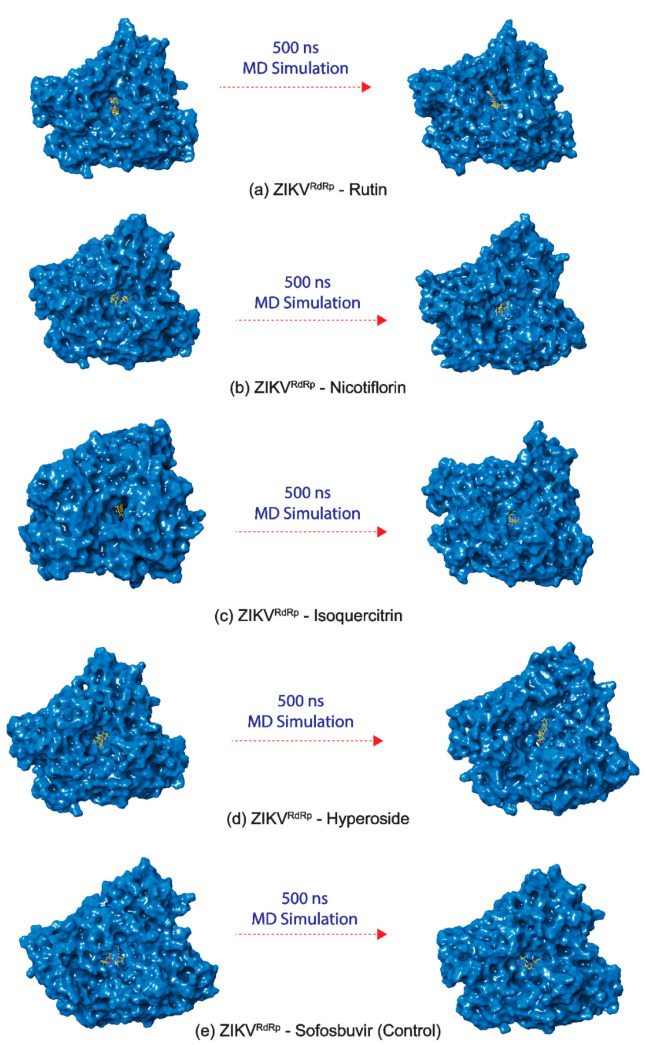
3D docked poses of ZIKV^RdRp^−bioflavonoids, i.e., (**a**) ZIKV^RdRp^−Rutin, (**b**) ZIKV^RdRp^−Nicotiflorin, (**c**) ZIKV^RdRp^−Isoquercitrin, (**d**) ZIKV^RdRp^−Hyperoside, and reference complex poses, i.e., (**e**) ZIKV^RdRp^−Sofosbuvir inhibitor, exhibiting transition of docked poses through 500 ns MD simulation interval.

**Figure 7:**
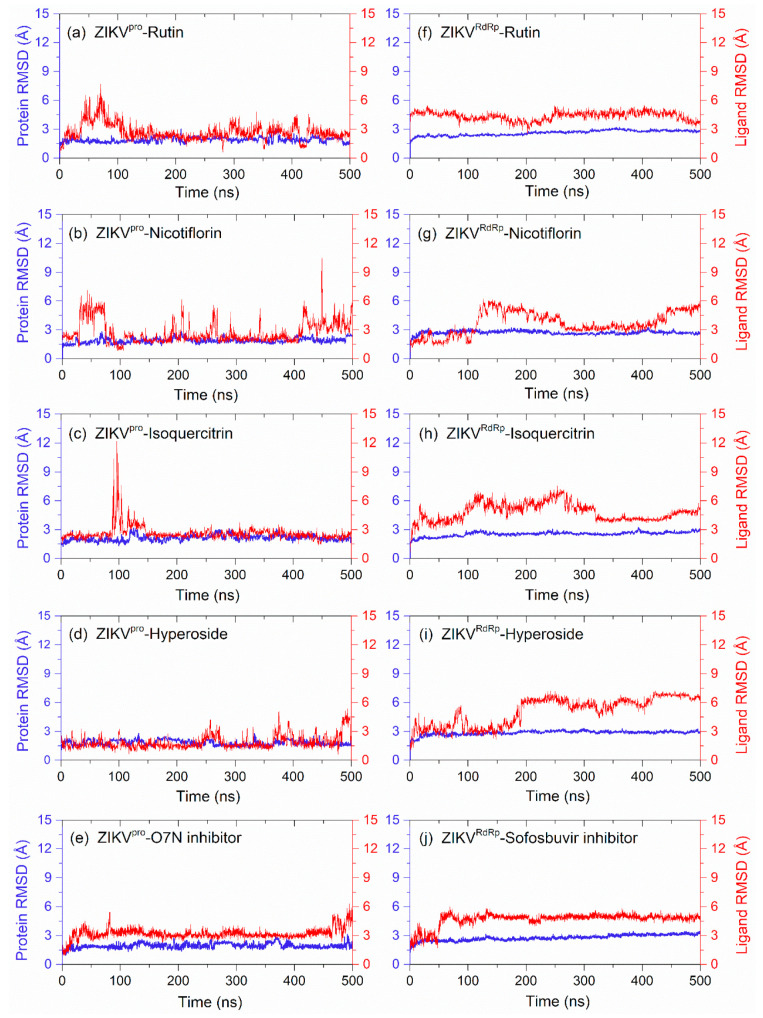
RMSD analysis on the docked viral proteins and ligands, i.e., bioflavonoids, (**a**) ZIKV^pro^−Rutin, (**b**) ZIKV^pro^−Nicotiflorin, (**c**) ZIKV^pro^−Isoquercitrin, (**d**) ZIKV^pro^−Hyperoside, (**f**) ZIKV^RdRp^−Rutin, (**g**) ZIKV^RdRp^−Nicotiflorin, (**h**) ZIKV^RdRp^−Isoquercitrin, and (**i**) ZIKV^RdRp^−Hyperoside, and reference compounds, (**e**) ZIKV^pro^−O7N inhibitor, and (**j**) ZIKV^RdRp^−Sofosbuvir inhibitor trajectories. Herein, protein RMSD values (blue color curves) were obtained based on Cα atoms of viral proteins while RMSD values for bioflavonoids or reference compounds (red curves) were extracted as ligand fit protein (Cα atoms) from the respective 500 ns MD simulation trajectories.

In this study, four common bioflavonoids, viz. Rutin, Nicotiflorin, Isoquercitrin, and Hyperoside, as bispecific inhibitors, showed substantial docking energy with targeted ZIKV proteins, and the resultant complexes; ZIKV^pro^−Rutin and ZIKV^RdRp^−Rutin, ZIKV^pro^−Nicotiflorin and ZIKV^RdRp^−Nicotiflorin, ZIKV^pro^−Isoquercitrin and ZIKV^RdRp^−Isoquercitrin, ZIKV^pro^−Hyperoside, and ZIKV^RdRp^−Hyperoside, were considered for 500 ns explicit MD simulation under constant pressure and temperature to analyze their dynamic stability and intermolecular interaction profiles with respect to time. Additionally, ZIKV^pro^−O7N inhibitor and ZIKV^RdRp^−Sofosbuvir inhibitor complexes were also studied under similar MD simulation conditions and marked as reference trajectories for comparative analysis with that of docked complexes of viral proteins with bioflavonoids (Figure 5, Figure 6 and Figure 7).

At first, stability and steadiness of docked bioflavonoids in the binding pocket of respective target proteins, i.e., ZIKV^pro^ and ZIKV^RdRp^, were observed at the end of 500 ns simulation by comparison to the respective initial frames, revealed the acceptable change in conformation of protein structure and deviations in docked ligands positions, similar to the respective reference inhibitors (Figure 5 and Figure 6). Additionally, analysis of intermolecular interaction profiles extracted for the last frames of viral protein-bioflavonoids and reference inhibitors were also found to maintain the several conserved molecular contacts by comparison to their respective initial poses (Table 1, Tables S4 and S5). Altogether, these observations suggested that docked bioflavonoids have substantially occupied the binding pocket of respective viral proteins by comparison to the reference inhibitors during the simulation interval. Hence, the generated 500 ns trajectories of the viral proteins docked with selected bioflavonoids were then analyzed for the statistical analysis, including root mean square deviation (RMSD), root mean square fluctuation (RMSF), and total interaction fraction for protein–ligand (PL) contact mapping by comparison to the respective reference trajectories as function of 500 ns interval.

#### 3.4.1. RMSD and RMSF Analysis

Initially, the protein and ligand RMSD values for the docked complexes of potential bioflavonoids with ZIKV^pro^ and ZIKV^RdRp^ were analyzed with respect to the initial pose as a reference frame (Figure 7). In each docked viral protein–bioflavonoids complexes, ZIKV^pro^ and ZIKV^RdRp^ showed considerable deviations (<3 Å) similar to the protein in respective reference trajectories, i.e., ZIKV^pro^−O7N inhibitor and ZIKV^RdRp^−Sofosbuvir inhibitor complexes. These observations were also supported by the respective RMSF values (<2.8 Å), except in the C-terminals (>5 Å) of proteins which can be ignored due to far distance from the binding pockets (Figure S1). Thus, RMSD analysis of viral proteins reveals no substantial effect of docked bioflavonoids on the global minima of the ZIKV proteins during 500 ns MD simulation interval.

Also, calculated RMSD values for all of the docked bioflavonoids in ZIKV^pro^ showed jumps to higher deviations; Rutin (<6 Å), Nicotiflorin (<10 Å), Isoquercitrin (<12 Å), and Hyperoside (<5 Å) by comparison to O7N inhibitor (<6 Å), followed by a state of steadiness during simulation interval. Similarly, higher deviations in the docked bioflavonoids were noticed with the ZIKV^RdRp^, Rutin (<5 Å), Nicotiflorin (<6 Å), Isoquercitrin (<7 Å), and Hyperoside (<7 Å) by comparison to Sofosbuvir inhibitor (<5 Å), was notice on some intervals followed by state of equilibrium till the end of the simulation. Interestingly, Rutin (<3 Å in ZIKV^pro^ and <4 Å in ZIKV^RdRp^) and Isoquercitrin (<3 Å in ZIKV^pro^ and <5 Å in ZIKV^RdRp^) were observed with most acceptable RMSD values and state of global minima by comparison to the reference inhibitors (<6 Å for both O7N inhibitor and Sofosbuvir inhibitor for ZIKV^pro^ and ZIKV^RdRp^, respectively) at the end of the 500 ns MD simulation interval. The observed RMSD results for the bioflavonoids and reference inhibitors of ZIKV proteins were supported by calculated RMSF values (<4 Å) as a function of simulation interval (Figure S2). Notably, higher deviations in both the docked bioflavonoids and reference inhibitors were suggested due to the interaction of heavy atoms in the ligands with the active residues in the binding pockets of the viral proteins that resulted in the conformational change in the binding poses of docked ligands, as observed in Figure 5 and Figure 6.

#### 3.4.2. Protein-Ligand Interaction Profiling

**Figure 8:**
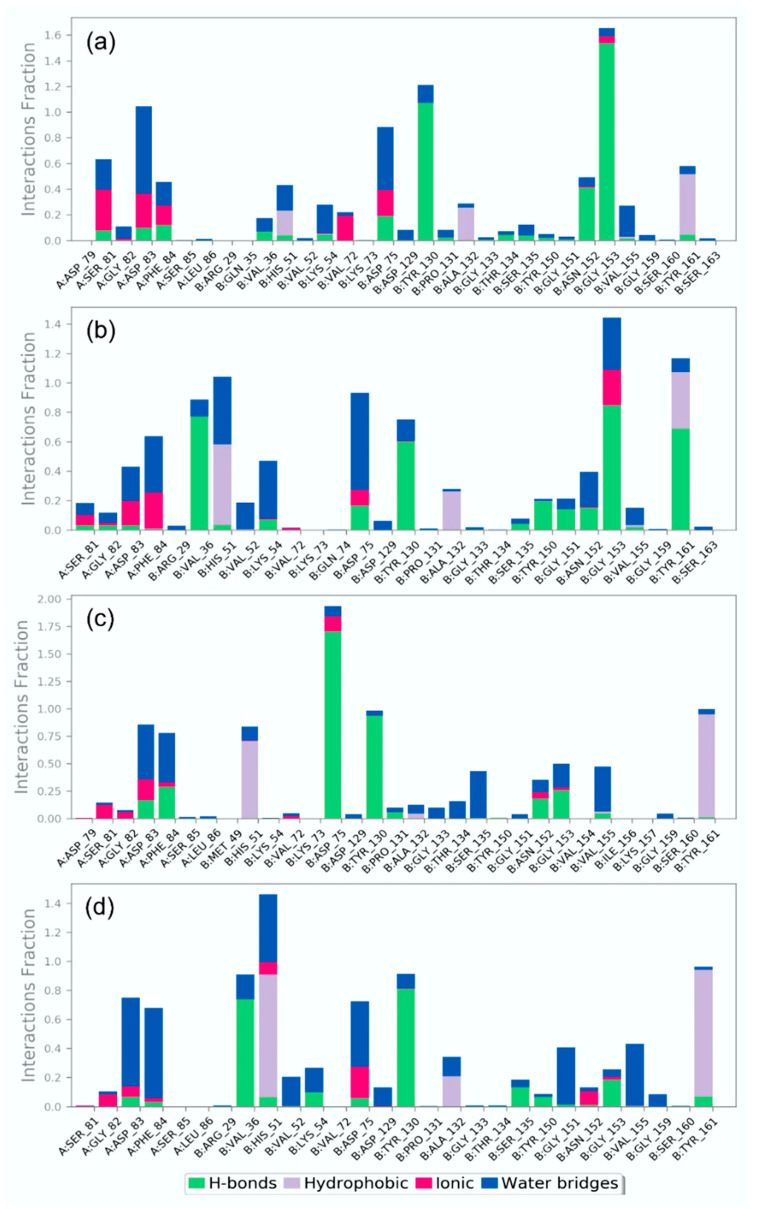
Protein-ligand interactions mapping for ZIKV^pro^ with selected bioflavonoids, i.e., (**a**) Rutin, (**b**) Nicotiflorin, (**c**) Isoquercitrin, and (**d**) Hyperoside, extracted from 500 ns MD simulations.

**Figure 9:**
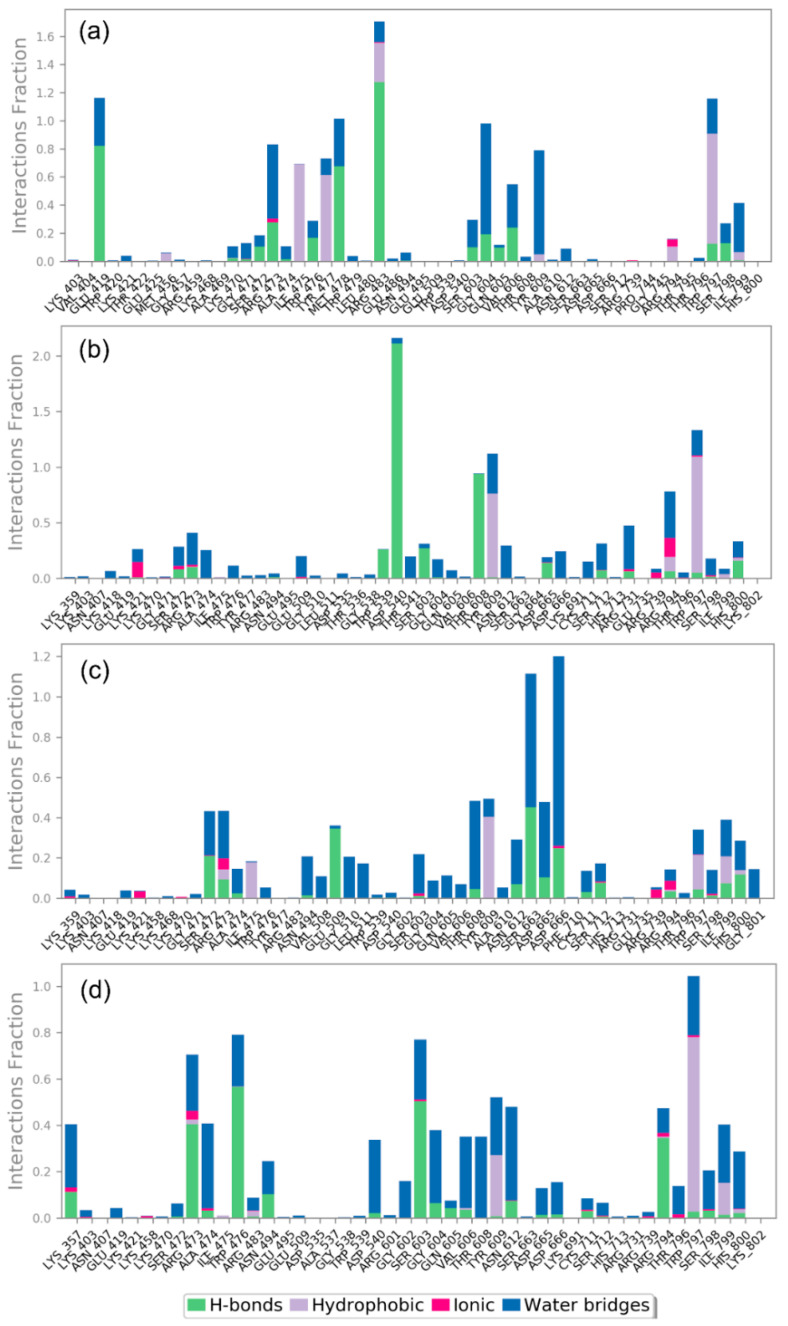
Protein-ligand interactions mapping for ZIKV^RdRp^ with selected bioflavonoids, i.e., (**a**) Rutin, (**b**) Nicotiflorin, (**c**) Isoquercitrin, and (**d**) Hyperoside, extracted from 500 ns MD simulations.

In the protein-ligand interaction, non-covalent interactions, especially H-bond and other interactions, such as hydrophobic interaction, ionic interactions, π-π stacking, salt bridges, and water bridges formation, have been reported as essential forces to maintain the stability to the complex. Therefore, in addition to the RMSD and RMSF analysis on the 500 ns simulation trajectory of each docked complex, protein-ligand contact profiling based on non-covalent interactions was also measured for all of the ZIKV^pro^−bioflavonoids and ZIKV^RdRp^−bioflavonoids complexes, and compared to the respective reference complexes, i.e., ZIKV^pro^−O7N inhibitor and ZIKV^RdRp^−Sofosbuvir inhibitor, respectively (Figure 8 and Figure 9, and Figures S3–S7).

In the case of ZIKV^pro^−ligand docked complexes, all of the selected bioflavonoids, i.e., Rutin, Nicotiflorin, Isoquercitrin, and Hyperoside, displayed significant intermolecular contact formation against reference O7N inhibitor (Figure 8). In comparison to the initial ZIKV^pro^−ligand docked complexes where the residues of ZIKV^pro^ (A:Ser^81^, A:Asp^83^, A:Phe^84^, B:Val^36^, B:His^51^, B:Asp^75^, B:Asn^152^, B:Gly^153^, and B:Tyr^161^) involved in the interaction with different bioflavonoid as ligands (Figure 3), the most of the residues were also noted in the protein-ligand contact maps obtained from 500 ns MD simulation trajectories, which suggested the stability of docked bioflavonoid in the binding pocket of ZIKV^pro^. Notably, B:His^51^ residue was observed for the formation of hydrophobic interaction for more than 50% of total interaction fraction in all ZIKV^pro^−bioflavonoid complexes except with Rutin (Figure 8). Similarly, B:TYR^161^ residue was observed to form hydrophobic interactions for more than 40% in ZIKV^pro^−Rutin and ZIKV^pro^−Nicotiflorin complexes while 90% in ZIKV^pro^−Isoquercitrin and ZIKV^pro^−Hyperoside complexes of total interaction fraction. Also, B:Gly^153^ was observed to form mainly H-bond in the ZIKV^pro^−Rutin and ZIKV^pro^−Nicotiflorin complexes only, and B:Tyr^130^ appeared as a naïve interacting residue in all of the ZIKV^pro^−bioflavonoids complexes during the simulation interval. Moreover, other essential residues, such as A:Ser^81^, A:Asp^83^, A:Phe^84^, B:Val^72^, B:Asp^75^, and B:Gly^153^, were observed to form ionic interactions for a shorter period in all of the ZIKV^pro^−bioflavonoids complexes; these ionic interactions might be crucial for the stability of ligands in the active binding pocket of viral protein (Figure 8). Besides, a few interacting residues, such as A:Asp^83^, B:Asp^75^, B:Tyr^130^, B:Gly^153^, and B:Tyr^161^, were observed as common in the intermolecular interactions of the ZIKV^pro^−bioflavonoids and reference ZIKV^pro^−O7N docked complex (Figure S3). Particularly, in the reference docked ZIKV^pro^−O7N complex, A:Asp^83^ was observed to form three H-bonds with the ligand for more than 100%, A:Ser^81^, A:Asp^83^, B:Asp^75^, B:Asp^129^, B:Tyr^130^, B:Gly^153^, and B:Tyr^161^ formed single H-bond for more than 50% of the total interaction fraction calculated as function of 500 ns simulation interval. Also, B:Val^155^ and B:Tyr^161^ residues showed hydrophobic interactions for more than 50% of the 500 ns simulation time (Figure S3). Additionally, contribution of water bridge formation was also noted in all of the ZIKV^pro^ complexes with the docked bioflavonoids and reference inhibitor (Figure 8 and Figure S3).

Furthermore, the analysis of protein-ligand contacts profiling for ZIKV^RdRp^−bioflavonoids docked complex also reveals substantial non-covalent interactions with conserved residues during simulation interval (Figure 9). In ZIKV^RdRp^−Rutin docked complex, Glu^419^, Gly^604^, Trp^797^, and Ser^798^ residues were continue interactions along with the additional residues such as Ile^475^ formed hydrophobic interaction (>75%), Arg^483^ formed H-bond (>100%) and hydrophobic interaction (~30%) of total interaction fraction during simulation. In ZIKV^RdRp^−Nicotiflorin complex, Trp^539^, Asp^540^, and Asp^665^ persist interactions fractions with the ligand along with Thr^608^ (H-bond for >90%), Tyr^609^ (hydrophobic interactions >75%), and Trp^797^ (hydrophobic interaction > 100%) during simulation. In ZIKV^RdRp^−Isoquercitrin docked complex, Asp^665^, Asp^666^, and Ile^799^ residues showed substantial contribution in total interaction fraction along with additional residues, such as Ser^472^ (H-bond for >20%), Glu^509^ (hydrophobic interaction > 30%), Tyr^609^ (hydrophobic interaction > 40%), and Ser^663^ (H-bond > 40%) during 500 ns interval. Also, in ZIKV^RdRp^−Hyperoside complex, the conserved residues, i.e., Asp^540^, and Asp^666^, were noticed for substantial interaction for shorter interval. However, this complex developed some stronger interactions with other residues, such as Arg^473^ and Arg^794^ (H-bond > 40%), Trp^476^ and Ser^603^ (H-bond > 55%), and Trp^797^ (hydrophobic interaction > 75%) of total interaction fraction during MD simulation. However, in the ZIKV^RdRp^-Sofosbuvir docked complex, Arg^473^, Thr^796^, Trp^797^, and Ser^798^ residues were substantially observed to form hydrogen bonds while Leu^736^ and Arg^739^ residues were noted for hydrophobic interactions (Figure S3). Moreover, all of the ZIKV^RdRp^ docked complexes exhibited water bridge formation for substantial fraction of total interaction during simulation interval (Figure 9 and Figure S3). Additionally, intermolecular interactions at 30% of the total simulation and the total number of residues contacts as function of 500 ns interval during simulation were also extracted for both ZIKV^pro^ and ZIKV^RdRp^ (Supplementary Figures S3–S7). Collectively, protein−ligand contact mapping suggests the stability of docked complexes, essentially contributed by the formation of H-bonds and hydrophobic interactions during 500 ns MD simulations.

### 3.5. Endpoint Free Binding Energy Calculation

**Figure 10:**
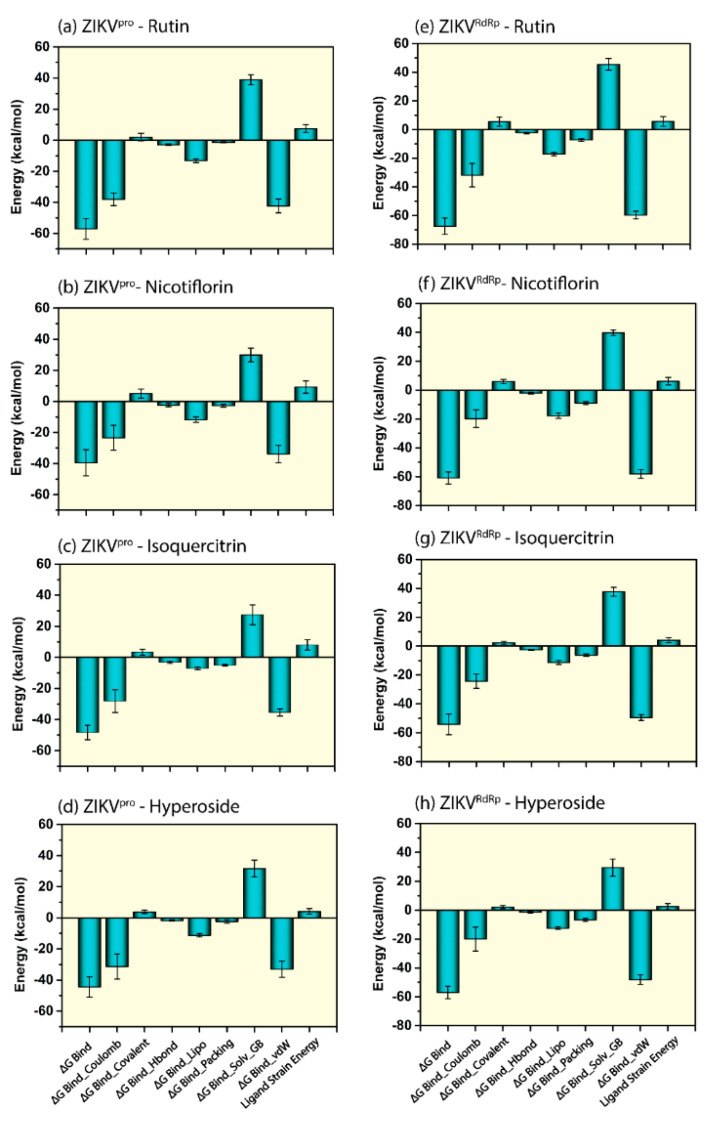
Calculated net MM/GBSA binding free energy (kcal/mol and energy dissociation components values (kcal/mol) with standard deviation values for extracted snapshots of docked complexes, i.e., (**a**) ZIKV^pro^−Rutin, (**b**) ZIKV^pro^−Nicotiflorin, (**c**) ZIKV^pro^−Isoquercitrin, (**d**) ZIKV^pro^−Hyperoside, (**e**) ZIKV^RdRp^−Rutin, (**f**) ZIKV^RdRp^−Nicotiflorin, (**g**) ZIKV^RdRp^−Isoquercitrin, and (**h**) ZIKV^RdRp^−Hyperoside from respective 500 ns MD simulation trajectories.

Molecular Mechanics Generalized Born Surface Area (MM/GBSA) method was applied to calculate the net binding free energy on the extracted poses from the last 10 ns MD simulation trajectory of respective docked complexes. Besides, energy dissociation components were also calculated to predict their contribution to the net stability of identified potential bioflavonoids complexes with viral proteins, i.e., ZIKV^pro^ and ZIKV^RdRp^. The analysis of net binding free energy for the screened bioflavonoids docked with the ZIKV^pro^ and ZIKV^RdRp^ showed considerable energy values by comparison to the respective reference complexes. Interestingly, Rutin docked with the ZIKV^pro^ and ZIKV^RdRp^ showed the highest negative free binding energy compared to other identified bioflavonoids (Table S8, Figure 10). Notably, Rutin was also identified for the formation of stable complex from 500 ns MD simulation via strong intermolecular interactions (Figure 7, Figure 8 and Figure 9).

By compared to the net binding free energy, i.e., −74.37 ± 6.44 kcal/mol of reference ZIKV^pro^−O7N complex, all of the ZIKV^pro^−bioflavonoids docked complexes showed less binding free energy but in the considerable range (Supplementary Table S8 and Figure 10). In contrast, the binding free energy of ZIKV^RdRp^−Rutin and ZIKV^RdRp^−Nicotiflorin docked complexes showed higher values, whereas ZIKV^RdRp^-Isoquercitrin, and ZIKV^RdRp^-Hyperoside showed less but close to the binding free energy of ZIKV^RdRp^−Sofosbuvir inhibitor complex (−59.83 ± 3.85 kcal/mol). In addition, the dissociation energy components were also calculated for all of the docked complexes, where *Δ*G*_Bind Lipo_*, and *Δ*G*_Bind vdW_* contributed to the complex stability, whereas *Δ*G*_Bind Solv GB_* was responsible for destabilizing the respective complexes. (Figure 10 and Figure S8 and Supplementary Table S8). Conclusively, these results suggested that the affinity and stability of ZIKV^pro^ were higher for Rutin, followed by Isoquercitrin, Hyperoside, and Nicotiflorin. In contrast, the stability and affinity of ZIKV^RdRp^ were higher with Rutin followed by Nicotiflorin, Hyperoside, and Isoquercitrin. Hence, net binding free energy calculated using MMGBSA analysis supports the screened four identified common bioflavonoids as putative inhibitors of viral proteins, viz. ZIKV^pro^ and ZIKV^RdRp^.

## 4. Conclusions

The essential role of ZIKV^pro^ and ZIKV^RdRp^ in polyprotein processing and genome replication of ZIKV as well as lack of respective homologs in humans, marks these viral proteins as molecular targets for the development of anti-ZIKV therapeutics. *Azadirachta indica* plant has placed itself in a category of natural resources with the best medicinal values. Therefore, this study evaluated the reported bioflavonoids from *Azadirachta indica* for their potential and therapeutic activity against the ZIKV^pro^ and ZIKV^RdRp^ domain using molecular docking simulations, drug-likeness, molecular dynamics simulation, and endpoint binding free energy calculations. Recently, the application of flavonoids as promising source of anti-ZIKV compounds were discussed to exert antiviral activity [92]. Notably, all of the selected bioflavonoids, i.e., Rutin, Nicotiflorin, Isoquercitrin, and Hyperoside, among 44 bioactive compounds against the ZIKV^pro^ and ZIKV^RdRp^ as bispecific inhibitors exhibit considerable binding affinity and dynamic stability. The screened compounds occupied the binding pockets via hydrogen and hydrophobic interactions along with π-π interactions with the essential residues of ZIKV^pro^ and ZIKV^RdRp^ against respective reference inhibitors. The analysis from MD simulation concluded that Rutin and Isoquercitrin with minimum deviation was more stable, followed by Hyperoside and Nicotiflorin with both the viral proteins, i.e., ZIKV^pro,^ and ZIKV^RdRp^. At last, end-point binding free energy calculation supports the Rutin as potent bispecific inhibitor of ZIKV^pro,^ and ZIKV^RdRp^. Overall, the present suggested the predicted bioflavonoids from Azadirachta indica as hit candidates and further accurate experimental validation is required to assess their potential as bispecific inhibitors of ZIKV^pro^, and ZIKV^RdRp^ for the treatment of ZIKV infection.

## Figures and Tables

**Table 1:** Redocking score and intermolecular interactions noted for the screened compounds with the viral proteins, i.e., ZIKV^pro^ and ZIKV^RdRp^, within 4 Å around the docked ligand in the respective binding pockets.

S. No.	Compounds	Redocking Score (kcal/mol)		H-Bond		* π-Cation Stacking/^†^ π-π Stacking/^‡^ Salt Bridge		Hydrophobic	
		ZIKV^pro^	ZIKV^RdRp^	ZIKV^pro^	ZIKV^RdRp^	ZIKV^pro^	ZIKV^RdRp^	ZIKV^pro^	ZIKV^RdRp^
1.	Rutin	−10.645	−11.038	A:Ser^81^, B:Val^36^, B:Asn^152^, B:Gly^153^	Glu^419^, Gly^604^, Asp^666^, Ser^798^, Ilu^799^	* B:His^51^	^†^ Trp^797^	A:Phe^84^, B:Val^36^, B:Trp^50^, B:Val^52^, B:Tyr^130^, B:Ala^132^, B:Tyr^150^, B:Val^154^, B:Tyr^161^	Ile^475^, Tyr^477^, Val^606^, Tyr^609^, Cys^711^, Trp^797^, Ile^799^
2.	Nicotiflorin	−9.986	−10.593	A:Ser^81^, B:Val^36^, B:Asn^152^, B:Gly^153^, B:Tyr^161^	Trp^539^, Asp^540^, Asp^665^, Asp^666^, Cys^711^	* B:His^51^	--	A:Phe^84^, B:Val^36^, B:Trp^50^, B:Val^52^, B:Tyr^130^, B:Ala^132^, B:Tyr^150^, B:Val^154^, B:Tyr^161^	Ala^474^, Trp^539^, Tyr^609^, Cys^711^, Trp^797^, Ile^799^
3.	Isoquercitrin	−8.666	−8.877	A:Asp^83^, A:Phe^84^, B:Asn^152^, B:Gly^153^	Asp^540^, Asp^665^, Asp^666^, Cys^711^, Ilu^799^	^†^ B:Tyr^161^	--	A:Phe^84^, B:Tyr^130^, B:Pro^131^, B:Ala^132^, B:Tyr^150^, B:Val^154^, B:Val^155^, B:Tyr^161^	Trp^539^, Tyr^609^, Cys^711^, Trp^797^, Ile^799^
4.	Hyperoside	−8.4	−7.907	A:Asp^83^, B:Val^36^, B:Asp^75^, B:Tyr^150^, B:Gly^153^	Asp^540^, Asp^666^	* B:His^51^, ^†^ B:Tyr^161^	--	A:Phe^84^, B:Val^36^, B:Val^52^, B:Tyr^130^, B:Ala^132^, B:Tyr^150^, B:Val^154^, B:Val^155^, B:Tyr^161^	Ile^475^, Val^606^, Tyr^609^, Cys^711^, Trp^797^, Ile^799^
5.	O7N (ZIKV^pro^ reference inhibitor)	−6.629	--	A:Asp^83^, B:Gly^153^, B:Tyr^161^	--	^‡^ A:Asp^83^, ^‡^ B:Asp^75^	--	A:Phe^84^, B:Trp^50^, B:Ala^132^, B:Tyr^150^, B:Val^154^, B:Val^155^, B:Tyr^161^	--
6.	Sofosbuvir (ZIKV^RdRp^ reference inhibitor)	--	−6.033	--	Asn^612^, Asp^665^, Arg^731^, Arg^739^, Thr^796^, Trp^797^, Ser^798^	--	--	--	Leu^513^, Tyr^609^, Cys^711^, Leu^736^, Tyr^760^, Met^763^, Tyr^768^, Trp^797^, Ile^799^

Symbols; * = π-Cation Stacking, † = π-π Stacking, and ‡ = Salt Bridge, stands for the interactions by respective marked residue.

## Data Availability

The datasets used and/or analyzed during the current study are available from the corresponding author on reasonable request.

## Supplementary Materials

The following supporting information can be downloaded at: https://www.mdpi.com/article/10.3390/molecules27082562/s1, Table S1: List of bioflavonoids reported in Azadirachta indica, used for structure based virtual screening against ZIKVpro and ZIKVRdRp, Table S2: List of virtually screened bioflavonoids from Azadirachta indica against ZIKVpro, Table S3: List of virtually screened bioflavonoids from Azadirachta indica against ZIKVRdRp, Table S4: Intermolecular interactions noted for the screened compounds with the viral proteins, i.e., ZIKVpro and ZIKVRdRp, within 4 Å around the docked ligand in the respective binding pockets, Table S5: List of various interactions and interacting residues in the active pocket of ZIKVpro and ZIKVRdRp with the selected bioflavonoids were logged from the last pose of respective 500 ns MD trajectories, Table S6: ADME profiling for the selected bioflavonoids from Azadirachta indica as inhibitor against ZIKVpro and ZIKVRdRp obtained from the swissADME online server (http://www.swissadme.ch/), Table S7: ADMET profiling for the selected bioflavonoids from Azadirachta indica as inhibitor against ZIKVpro and ZIKVRdRp obtained from the admetSAR online server (http://lmmd.ecust.edu.cn/admetsar2/), Table S8: Calculated energy components and net binding free energies (kcal/mol) values for ZIKVpro and ZIKVRdRp complex with selected bioflavonoids against reference compound snapshots collected from the respective 500 ns MD simulation trajectories, Figure S1: RMSF values plotted for alpha carbon atoms of ZIKVpro and ZIKVRdRp docked with selected bioflavonoids i.e., (a,b) Rutin, (c,d) Nicotiflorin, (e,f) Isoquercitrin, (g,h) Hyperoside, and as well as the reference inhibitors (i) O7N (ZIKVpro reference inhibitor) and (j) Sofosbuvir (ZIKVRdRp reference inhibitor), were extracted from the respective 500 ns MD simulation interval, Figure S2: RMSF values plotted for the bioflavonoids in the docked complexes, i.e., (a) ZIKVpro-Rutin, (b) ZIKVpro-Nicotiflorin, (c) ZIKVpro-Isoquercitrin, (d) ZIKVpro-Hyperoside, (e) ZIKVpro-O7N (Control), (f) ZIKVRdRp-Rutin, (g) ZIKVRdRp-Nicotiflorin, (h) ZIKVRdRp-Isoquercitrin, (i) ZIKVRdRp-Hyperoside, (j) ZIKVRdRp-Sofosbuvir (Control), fit with protein extracted from the respective 500 ns MD simulation interval, Figure S3: Protein-ligand interactions mapping for reference docked complexes, i.e., (a,b) ZIKVpro–O7N, (c,d) ZIKVRdRp–Sofosbuvir inhibitor, extracted from 500 ns MD simulations. In 2D interaction diagram, the residues tyrosine, Valine, and Phenylalanine (green), Aspartic acid (red), histidine and asparagine (blue), and glycine (grey) exhibit the hydrophobic, negative, polar, and non-polar interactions, respectively along with hydrogen bonding (pink arrow) and pi-pi stacking (green line) with the receptor are extracted at 30% of the total MD simulation interaction interval, Figure S4: The panel shows which residues of ZIKVpro interaction with the selected ligands i.e., a. Rutin, b. Nicotiflorin, c. Isoquercitrin, and d. Hyperoside, in each trajectory frame, over the course of the 500 ns md trajectory. Some residues make more than one specific contact with the ligand, which is represented by a darker shade of orange, according to the scale to the right of the plot, Figure S5: The panel shows which residues of ZIKVRdRp interact with the selected ligands, i.e., a. Rutin, b. Nicotiflorin, c. Isoquercitrin, and d. Hyperoside, in each trajectory frame, over the course of the 500 ns md trajectory. Some residues make more than one specific contact with the ligand, which is represented by a darker shade of orange, according to the scale to the right of the plot, Figure S6: 2D interaction diagram of protein-ligand interactions maps for ZIKVpro with selected bioflavonoids, i.e., (a) Rutin, (b) Nicotiflorin, (c) Isoquercitrin, and (d) Hyperoside extracted from the total 500 ns MD simulations. Herein, residues tyrosine, Valine, and Phenylalanine (green), Aspartic acid (red), histidine and asparagine (blue), and glycine (grey) exhibit the hydrophobic, negative, polar, and non-polar interactions, respectively, along with hydrogen bonding (pink arrow) and π-π stacking (green line) with the receptor are extracted at 30% of the total MD simulation interaction interval, Figure S7: 2D interaction diagram of protein-ligand interactions mapping for ZIKVRdRp with selected natural compounds, i.e., (a) Rutin, (b) Nicotiflorin, (c) Isoquercitrin, and (d) Hyperoside, extracted from 500 ns MD simulations. Residues tyrosine, Valine, and Phenylalanine (green), Aspartic acid (red), histidine and asparagine (blue), and glycine (grey) exhibit the hydrophobic, negative, polar, and non-polar interactions, respectively, along with hydrogen bonding (pink arrow) and pi-pi stacking (green line) with the receptor are extracted at 30% of the total MD simulation interaction interval, Figure S8: Calculated free energy components and net MM/GBSA binding free energy (kcal/mol) with standard deviation values for extracted snapshots of reference docked complexes, i.e., (a). ZIKVpro-O7N, (b). ZIKVRdRp–Sofosbuvir, from respective 500 ns MD simulation trajectories.

## Sample Availability

Not available.
