# Supplementary material for: Discovery of Bispecific Lead Compounds from Azadirachta indica against ZIKA NS2B-NS3 Protease and NS5 RNA Dependent RNA Polymerase Using Molecular Simulations
Source: Molecules. 2022 Apr 15;27(8):2562. doi: 10.3390/molecules27082562 (PMC9025849; doi:10.3390/molecules27082562)
Supplement: Supplementary file 1 [file molecules-27-02562-s001.zip › molecules-1670099-supplementary.pdf]

Discovery of bispecific lead compounds from *Azadirachta indica* against ZIKA NS2B-NS3 Protease and NS5 RNA dependent RNA polymerase using molecular simulations

Sanjay Kumar [1,2], Sherif A. El-Kafrawy [3,4], Shiv Bharadwaj [5*], S. S. Maitra[1], Thamir A. Alandijany [3,4], Arwa A. Faizo [3,4], Aiah M. Khateb [3,6], Vivek Dhar Dwivedi [2*], Esam I. Azhar [3,4*]

[1] School of Biotechnology, Jawaharlal Nehru University, New Delhi 110067, INDIA.
[2] Center for Bioinformatics, Computational and Systems Biology, Pathfinder Research and Training Foundation, Greater Noida 201308, India.
[3] Special Infectious Agents Unit, King Fahd Medical Research Center, King Abdulaziz University, PO Box 128442, Jeddah 21362, Saudi Arabia,
[4] Department of Medical Laboratory Technology, Faculty of Applied Medical Science, King Abdulaziz University, Jeddah 21589, Saudi Arabia.
[5] Laboratory of Ligand Engineering, Institute of Biotechnology of the Czech Academy of Sciences, BIOCEV Research Center, Vestec, Czech Republic.
[6] Medical Laboratory Technology Department, College of Applied Medical Sciences, Taibah University, Madinah, Saudi Arabia, Medina 42353, KSA.
* Correspondence: Esam I Azhar; Email: eazhar@kau.edu.sa; Shiv Bharadwaj; Email: shiv.bharadwaj@ibt.cas.cz; Vivek Dhar Dwivedi; Email vivek_bioinformatics@yahoo.com

Results and Discussion

S1.1 Structure based virtual screening

Table S1. List of bioflavonoids reported in *Azadirachta indica,* used for structure based virtual screening against $ZIKV^{pro}$ and $ZIKV^{RdRp.}$

S. No.	Natural compounds
**1**	Azadirachtin
**2**	Azadirachtol
**3**	Azadiradione
**4**	Behenic acid
**5**	Beta-sitosterol
**6**	Catechin
**7**	Desacetylnimbin
**8**	Desacetylsalannin
**9**	Epicatechin
**10**	Gedunin
**11**	Hyperoside
**12**	Isomeldenin
**13**	Isonimbinolide
**14**	Kaempferol
**15**	Kaempferoodglucoside
**16**	Kaempferol-3-O-rutinoside
**17**	khivorin
**18**	Kulactone
**19**	Limocinin
**20**	Margocinin
**21**	Margolone
**22**	Nimbandiol
**23**	Nimbidiol
**24**	Nimbin
**25**	Nimbinin
**26**	Nimbinone
**27**	Nimbiol
**28**	Nimbione
**29**	Nimbolide
**30**	Nimocinol
**31**	Nimosone
**32**	Ohchinin
**33**	Acetate
**34**	Ohchinolideb
**35**	Quercetin
**36**	Isoquercitrin
**37**	Rutin
**38**	Salannin
**39**	Salannol
**40**	Salannolide
**41**	Scopoletin
**42**	Sugiol
**43**	Vilasinin
**44**	Zafaral

Table S2. List of virtually screened bioflavonoids from *Azadirachta indica* against $ZIKV^{pro}$

S. No.	Inhibitors of ZIKA Protease	Docking score (kcal/mol)
1	Rutin	-10.616
2	Kaempferol-3-O-rutinoside	-9.957
3	Isoquercitrin	-8.637
4	Hyperoside	-8.372
5	Kaempferol-O-D-glucoside	-7.539
6	Epicatechin	-7.1
7	Quercetin	-6.619
8	Myricetin	-6.493
9	Catechin	-6.425
10	Kaempferol-3-O-rutinoside	-6.29
11	Hyperoside	-6.048
12	Kaempferol	-4.963
13	Margocinin	-4.29
14	Scopoletin	-3.853
15	Nimocinol	-3.832
16	Nimbinone	-3.606
17	Nimbiol	-3.554
18	nimbione	-3.554
19	Nimosone	-3.447
20	Sugiol	-3.446
21	Zafaral	-2.9

Table S3. List of virtually screened bioflavonoids from *Azadirachta indica* against ZIKV^{RdRp}

S. No.	**Inhibitors of ZIKA^{RdRp}**	**Docking score (kcal/mol)**
**1**	Rutin	-11.01
**2**	Kaempferol-3-O-rutinoside	-10.564
**3**	Rutin	-10.428
**4**	Isoquercitrin	-8.848
**5**	Myricetin	-8.168
**6**	Hyperoside	-7.878
**7**	Kaempferol-O-D-glucoside	-6.642
**8**	Isonimbinolide	-6.077
**9**	Quercetin	-6.06
**10**	desacetylnimbin	-6.046
**11**	Catechin	-5.993
**12**	Scopoletin	-5.694
**13**	Nimbin	-5.666
**14**	SALANNOLIDE	-5.419
**15**	Margocinin	-5.126
**16**	Ohchinolide B	-5.021
**17**	Epicatechin	-5.009
**18**	Nimosone	-4.961
**19**	Nimbinone	-4.746
**20**	Kaempferol	-4.691
**21**	Nimbiol	-4.539

Table S4: Intermolecular interactions noted for the screened compounds with the viral proteins, i.e., ZIKV^{pro} and ZIKV^{RdRp}, within 4 Å around the docked ligand in the respective binding pockets

S. no.	Compounds	Polar		Negative		Positive		Glycine	
		ZIKV^{pro}	ZIKV^{RdRp}	ZIKV^{pro}	ZIKV^{RdRp}	ZIKV^{pro}	ZIKV^{RdRp}	ZIKV^{pro}	ZIKV^{RdRp}
1.	Rutin	A:Ser^{81}, B:Ser^{135}, B:Asn^{152}	Gln^{605}, Ser^{663}, Ser^{712}, Ser^{798}, His^{800}	B:Asp^{75}, A:Asp^{83}, B:Asp^{129}	Glu^{419}, Asp^{665}, Asp^{666}	B:His^{51}, B:Lys^{54}	Lys^{470}, Arg^{473}, Arg^{483}, Arg^{739}	A:Gly^{82}, B:Gly^{133}, B:Gly^{151}, B:Gly^{153}	Gly^{604}
2.	Nicotiflorin	A:Ser^{81}, B:Ser^{135}, B:Asn^{152}	Ser^{472}, Thr^{536}, Thr^{541}, Ser^{603}, Thr^{608}, Asn^{612}, Ser^{663}, Ser^{798}, His^{800}	A:Asp^{83}, B:Asp^{75}, B:Asp^{129}	Asp^{540}, Asp^{665}, Asp^{666}	B:His^{51}, B:Lys^{54}	Arg^{473}, Arg^{601}, Lys^{691}	A:Gly^{82}, B:Gly^{133}, B:Gly^{151}, B:Gly^{153}	Gly^{538}, Gly^{604}, Gly^{664}
3.	Isoquercitrin	A:Ser^{85}, B:Thr^{134}, B:Ser^{135}, B:Asn^{152}	Ser^{603}, Thr^{608}, Asn^{612}, Ser^{663}, Ser^{712}, Ser^{798}, His^{800}	A:Asp^{83}, B:Asp^{75}, B:Asp^{129}	Asp^{540}, Asp^{665}, Asp^{666}	B:His^{51}	-	A:Gly^{82}, B:Gly^{151}, B:Gly^{153}	Gly^{604}, Gly^{664}
4.	Hyperoside	B:Thr^{134}, B:Ser^{135}	Ser^{603}, Gln^{605}, Thr^{608}, Asn^{612}, Ser^{663}, Ser^{798}, His^{800}	A:Asp^{83}, B:Asp^{75}, B:Asp^{129}	Asp^{540}, Asp^{665}, Asp^{666}	B:His^{51}, B:Lys^{54}	Arg^{473}, Arg^{601}	A:Gly^{82}, B:Gly^{133}, B:Gly^{151}, B:Gly^{153}	Gly^{604}
5.	O7N (control inhibitor for ZIKV^{pro})	A:Ser^{81}, B:Ser^{135}, B:Asn^{152}	-	A:Asp^{83}, B:Asp^{75}	-	B:His^{51}, B:Lys^{54}	-	A:Gly^{82}, B:Gly^{153}	-
6.	Sofosbuvir (control inhibitor for ZIKV^{RdRp})	-	Thr^{608}, Asn^{612}, Ser^{663}, Ser^{712}, His^{713}, Thr^{795}, Thr^{796}, Ser^{798}, His^{800}	-	Asp^{665}, Asp^{666}	-	Lys^{470}, Arg^{473}, Arg^{731}, Arg^{739}	-	Gly^{664}

Table S5. List of various interactions and interacting residues in the active pocket of ZIKV^{Pro} and ZIKV^{RdRp} with the selected bioflavonoids were logged from the last pose of respective 500 ns MD trajectories.

S. no.	Compounds	H- bond		*π-cation stacking/ †π-π stacking/ ‡Salt bridge		Hydrophobic		Polar		Negative		Positive		Glycine	
		ZIKV^{pro}	ZIKV^{RdRp}	ZIKV^{pro}	ZIKV^{RdRp}	ZIKV^{pro}	ZIKV^{RdRp}	ZIKV^{pro}	ZIKV^{RdRp}	ZIKV^{pro}	ZIKV^{RdRp}	ZIKV^{pro}	ZIKV^{RdRp}	ZIKV^{pro}	ZIKV^{RdRp}
1.	Rutin	B:Tyr^{130}, B:Tyr^{150}, B:Asn^{152}, B:Gly^{153}	Arg^{473}, Arg^{483}		†Trp^{797}	A:Phe^{84}, B:Tyr^{130}, B:Pro^{131} , B:Ala^{132}, B:Tyr^{150}, B:Val^{154}, B:Val^{155}, B:Tyr^{161}	Met^{456}, Ile^{475}, Trp^{476}, Tyr^{477}, Met^{478}, Val^{606}, Tyr^{609}, Trp^{797}, Ile^{799}	A:Ser^{81}, B:His^{51}, B:Thr^{134}, B:Ser^{135}, B:Asn^{152}	Ser^{472}, Ser^{603}, Gln^{605}, Thr^{608}, Ser^{798}	A:Asp^{83}	Glu^{419}	-	Arg^{473}, Arg^{483},	B:Gly^{133}, B:Gly^{151}, B:Gly^{153}	Gly^{471}, Gly^{604},
2.	Nicotiflorin	B:Tyr^{130}	Thr^{608}, Arg^{794}, Trp^{797}		†Trp^{797}	B:Val^{52}, B:Tyr^{130}, B:Pro^{131} , B:Ala^{132}, B:Val^{154}, B:Val^{155}, B:Tyr^{161}	Ile^{475}, Tyr^{477}, Tyr^{609}, Cys^{711}, Trp^{797},	B:His^{51}, B:Ser^{135}	Ser^{603}, Thr^{608}, Asn^{612}, Ser^{712}, Ser^{798}, His^{800},	B: Asp^{129}	Glu^{419}, Glu^{509}, Asp^{540}	-	Lys^{418}, Lys^{421}, Arg^{473}, Arg^{794}	B:Gly^{133},B:Gly^{153}	Gly^{604}
3.	Isoquercitrin	B:Asp^{75} B:Tyr^{130}	Glu^{509}, Ser^{663}, His^{800}	†B:His^{51}, †B:Tyr^{161}	†His^{800}, †Tyr^{609}	B:Met^{49}, B:Tyr^{130}, B:Pro^{131} , B:Ala^{132}, B:Tyr^{150}, B:Tyr^{161}	Val^{508}, Leu^{511}, Leu^{516}, Tyr^{609}, Cys^{711},Trp^{797}, , Ile^{799},	B:His^{51}, B:Thr^{134}, B:Ser^{135}, B:Asn^{152}	Thr^{608}, Asn^{612,} Ser^{663}, Ser^{712}, Ser^{798}, His^{800},	A: Asp^{83} B: Asp^{75}	Glu^{509}, Asp^{665} , Asp^{666,}	-	His^{497}, Arg^{473}	B: Gly^{151}	Gly^{510}, Gly^{664}
4.	Hyperoside	B:Tyr^{130}	Trp^{476}	†B:His^{51}, †B:Tyr^{161}	†Trp^{797}	A:Phe^{84}, B:Val^{52}, B:Tyr^{130}, B:Pro^{131} , B:Ala^{132}, B:Val^{154}, B:Val^{155}, B:Tyr^{161}	Ala^{474}, Ile^{475}, Trp^{476}, Val^{606}, Tyr^{609}, Trp^{797}, Ile^{799}	B:His^{51}, B:Ser^{135}, B:Asn^{152} ,	Ser^{603}, Thr^{608}, Asn^{494}, Ser^{798},	A:Asp^{83}		-	Lys^{403}, Arg^{473},	B:Gly^{151}, B:Gly^{153}	Gly^{604}
5.	O7N (Reference inhibitor for ZIKV^{pro})	B:His^{51,} B:Asp^{75}, B:Tyr^{130}, B:Gly^{151}, B:Gly^{153}, B:Tyr^{161}		‡B:Asp^{75}, ‡B:Asp^{129}		A:Phe^{84}, B:Val^{36}, B:Val^{52}, B:Val^{72}, B:Tyr^{130}, B:Pro^{131} , B:Ala^{132}, B:Val^{154}, B:Val^{155}, B:Tyr^{161}		B:His^{51}, B:Thr^{134}, B:Ser^{135}, B:Asn^{152} ,		A:Asp^{83} B:Asp^{75}, B:Asp^{129}		-		A:Gly^{82}, B:Gly^{133}, B:Gly^{151}, B:Gly^{153},	
6.	Sofosbuvir (Reference inhibitor for ZIKV^{RdRp})		Trp^{797}		*Arg^{739}		Leu^{513}, Tyr^{609}, Cys^{711}, Leu^{736}, Met^{763}, Leu^{767}, Tyr^{768}, Trp^{797}, Ile^{799}		Ser^{472}, Ser^{712}, His^{713}, Thr^{795}, Thr^{796}, Ser^{798}, His^{800},		Glu^{735}		Arg^{731}, Arg^{739}, Arg^{473},		

S1.2 ADMET analysis

Table S6. ADME profiling for the selected bioflavonoids from *Azadirachta indica* as inhibitor against $ZIKV^{pro}$ and $ZIKV^{RdRp}$ obtained from the swissADME online server (http://www.swissadme.ch/)

Properties	Rutin	Nicotiflorin	Isoquercitrin	Hyperoside	O7n	Sofosbuvir
ILOGP	2.43	2.79	2.11	2.11	1.75	3.23
XLOGP3	-0.33	0.02	0.36	0.36	-1.85	0.99
WLOGP	-1.69	-1.39	-0.54	-0.54	-3.92	1.75
MLOGP	-3.89	-3.43	-2.59	-2.59	-1.71	0.82
SILICOS-IT LOG P	-2.11	-1.64	-0.59	-0.59	-0.07	0.61
CONSENSUS LOG P	-1.12	-0.73	-0.25	-0.25	-1.16	1.48
ESOL LOG S	-3.3	-3.42	-3.04	-3.04	-2.09	-3.27
ESOL SOLUBILITY (MG/ML)	3.08E-01	2.24E-01	4.23E-01	4.23E-01	5.13E+00	2.86E-01
ESOL SOLUBILITY (MOL/L)	5.05E-04	3.76E-04	9.10E-04	9.10E-04	8.13E-03	5.41E-04
ESOL CLASS	Soluble	Soluble	Soluble	Soluble	Soluble	Soluble
ALI LOG S	-4.87	-4.81	-4.35	-4.35	-3.13	-4.11
ALI SOLUBILITY (MG/ML)	8.30E-03	9.31E-03	2.10E-02	2.10E-02	4.65E-01	4.14E-02
ALI SOLUBILITY (MOL/L)	1.36E-05	1.57E-05	4.51E-05	4.51E-05	7.38E-04	7.83E-05
ALI CLASS	Moderately soluble	Moderately soluble	Moderately soluble	Moderately soluble	Soluble	Moderately soluble
SILICOS-IT LOGSW	-0.29	-0.88	-1.51	-1.51	-7.14	-4.09
SILICOS-IT SOLUBILITY MG/ML)	3.15E+02	7.77E+01	1.43E+01	1.43E+01	4.58E-05	4.32E-02
SILICOS-IT SOLUBILITY (MOL/L)	5.15E-01	1.31E-01	3.08E-02	3.08E-02	7.26E-08	8.17E-05
SILICOS-IT CLASS	Soluble	Soluble	Soluble	Soluble	Poorly soluble	Moderately soluble
GI ABSORPTION	Low	Low	Low	Low	Low	Low
BBB PERMEANT	No	No	No	No	No	No
PGP SUBSTRATE	Yes	Yes	No	No	Yes	Yes
CYP1A2 INHIBITOR	No	No	No	No	No	No
CYP2C19 INHIBITOR	No	No	No	No	No	No
CYP2C9 INHIBITOR	No	No	No	No	No	No
CYP2D6 INHIBITOR	No	No	No	No	No	No
CYP3A4 INHIBITOR	No	No	No	No	No	Yes
LOG KP (CM/S)	-10.26	-9.91	-8.88	-8.88	-11.46	-8.83
LIPINSKI #VIOLATIONS	3	3	2	2	3	2
GHOSE #VIOLATIONS	4	4	1	1	4	1
VEBER #VIOLATIONS	1	1	1	1	1	2
EGAN #VIOLATIONS	1	1	1	1	1	1
MUEGGE #VIOLATIONS	4	3	3	3	3	2
BIOAVAILABILITY SCORE	0.17	0.17	0.17	0.17	0.17	0.17
PAINS #ALERTS	1	0	1	1	0	0
BRENK #ALERTS	1	0	1	1	2	1
LEADLIKENESS #VIOLATIONS	1	1	1	1	2	2
SYNTHETIC ACCESSIBILITY	6.52	6.48	5.32	5.32	6.66	6.02

Table S7. ADMET profiling for the selected bioflavonoids from *Azadirachta indica* as inhibitor against ZIKV^{pro} and ZIKV^{RdRp} obtained from the admetSAR online server (http://lmmd.ecust.edu.cn/admetsar2/).

Properties	Compounds					
	Rutin	Nicotiflorin	Isoquercitrin	Hyperoside	O7N	Sofosbuvir
Ames's toxicity	-	-	+	+	-	-
Acute Oral Toxicity (c)	III	III	III	III	III	III
Androgen receptor binding	+	+	+	+	+	+
Aromatase binding	+	+	+	+	+	+
Avian toxicity	-	-	-	-	-	-
Blood Brain Barrier	-	-	-	-	+	+
BRCP inhibitor	-	-	-	-	-	-
Biodegradation	-	-	-	-	-	-
BSEP inhibitor	+	+	-	-	+	+
Caco-2	-	-	-	-	-	-
Carcinogenicity (binary)	-	-	-	-	-	-
Carcinogenicity (trinary)	Non-required	Non-required	Non-required	Non-required	Non-required	Non-required
crustacea aquatic toxicity	-	-	-	-	-	+
CYP1A2 inhibition	-	-	-	-	-	-
CYP2C19 inhibition	-	-	-	-	-	-
CYP2C9 inhibition	-	-	-	-	-	-
CYP2C9 substrate	-	-	-	-	-	-
CYP2D6 inhibition	-	-	-	-	-	-
CYP2D6 substrate	-	-	-	-	-	-
CYP3A4 inhibition	-	-	-	-	-	+
CYP3A4 substrate	+	+	+	+	+	+
CYP inhibitory promiscuity	-	-	-	-	-	-
Eye corrosion	-	-	-	-	-	-
Eye irritation	-	-	-	-	-	-
Estrogen receptor binding	+	+	+	+	+	+
Fish aquatic toxicity	+	+	+	+	-	+
Glucocorticoid receptor binding	+	+	+	+	-	+
Honeybee toxicity	+	+	+	+	-	-
Hepatotoxicity	+	+	+	+	-	+
Human either-a-go-go inhibition	-	-	-	-	+	-
Human Intestinal Absorption	+	+	+	+	+	+
Human oral bioavailability	-	-	-	-	+	-
MATE1 inhibitor	-	-	-	-	-	-
micronuclear	+	+	+	+	+	+
Acute Oral Toxicity	2.593124151	2.144020319	3.130611181	3.130611181	2.557645798	3.940588474
OATP1B1 inhibitor	+	+	+	+	+	+
OATP1B3 inhibitor	+	+	+	+	+	+
OATP2B1 inhibitor	-	-	+	+	-	-
OCT1 inhibitor	-	-	-	-	-	-
OCT2 inhibitor	-	-	-	-	-	-
P-glycoprotein inhibitor	-	-	-	-	+	+
P-glycoprotein substrate	-	+	-	-	+	-
PPAR gamma	+	+	+	+	+	+
Plasma protein binding	0.982454121	0.907816708	0.891581595	0.891581595	0.722036541	1.100830078
Subcellular localization	Mitochondria	Mitochondria	Mitochondria	Mitochondria	Mitochondria	Mitochondria
Tetrahymena pyriformis	1.20004499	1.10743773	0.801442623	0.801442623	-0.038675837	0.250124514
Thyroid receptor binding	+	+	-	-	+	+
UGT catalyzed	+	+	+	+	-	-
Water solubility	-2.772404247	-2.772404247	-2.448882454	-2.448882454	-2.729500059	-3.746490711

S1.4 Molecular dynamics simulation analysis

S1.4.1 RMSD and RMSF analysis

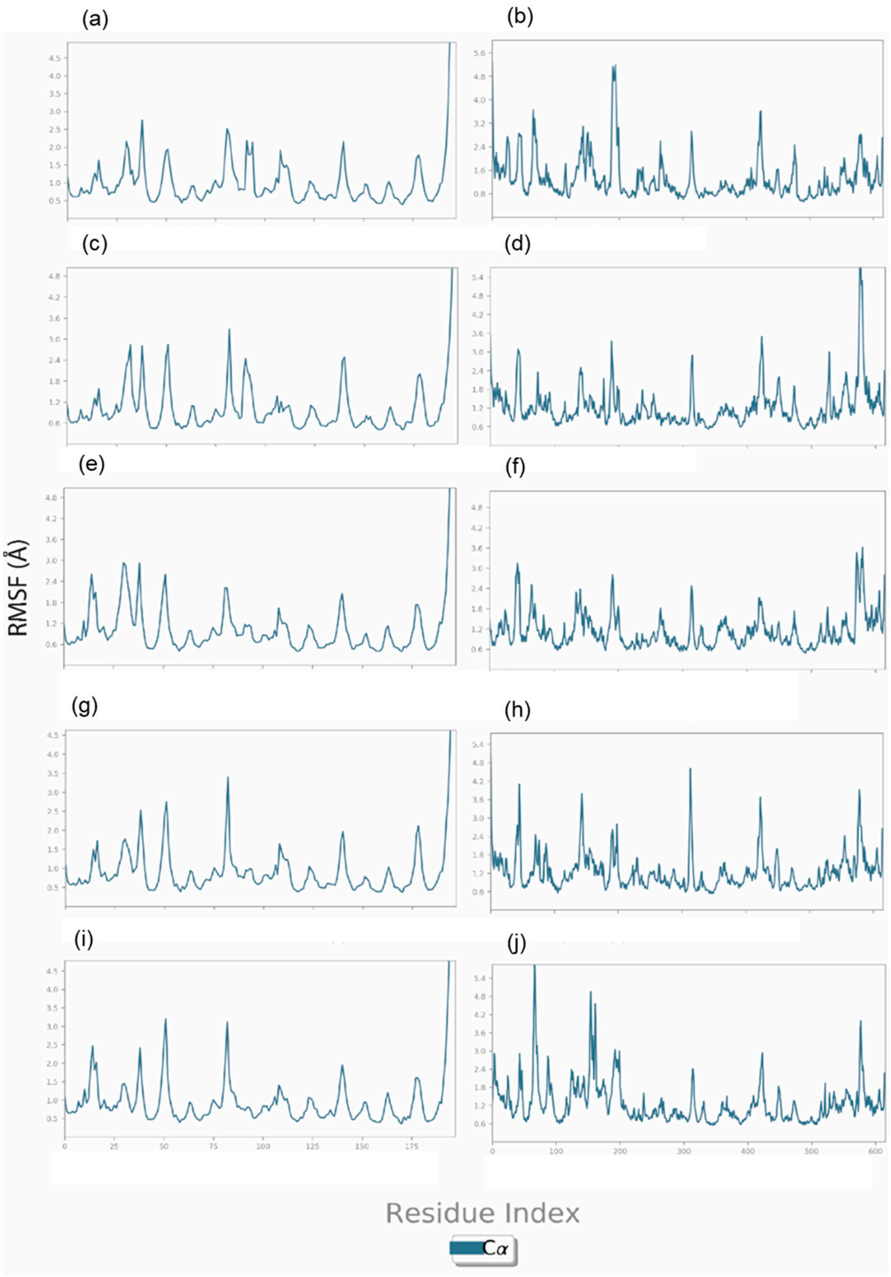

Figure S1. RMSF values plotted for alpha carbon atoms of ZIKV^{pro} and ZIKV^{RdRp} docked with selected bioflavonoids i.e., (a-b) Rutin, (c-d) Nicotiflorin, (e-f) Isoquercitrin, (g-h) Hyperoside, and as well as the reference inhibitors **(i)** O7N (ZIKV^{pro} reference inhibitor) and (j) Sofosbuvir (ZIKV^{RdRp} reference inhibitor), were extracted from the respective 500 ns MD simulation interval.

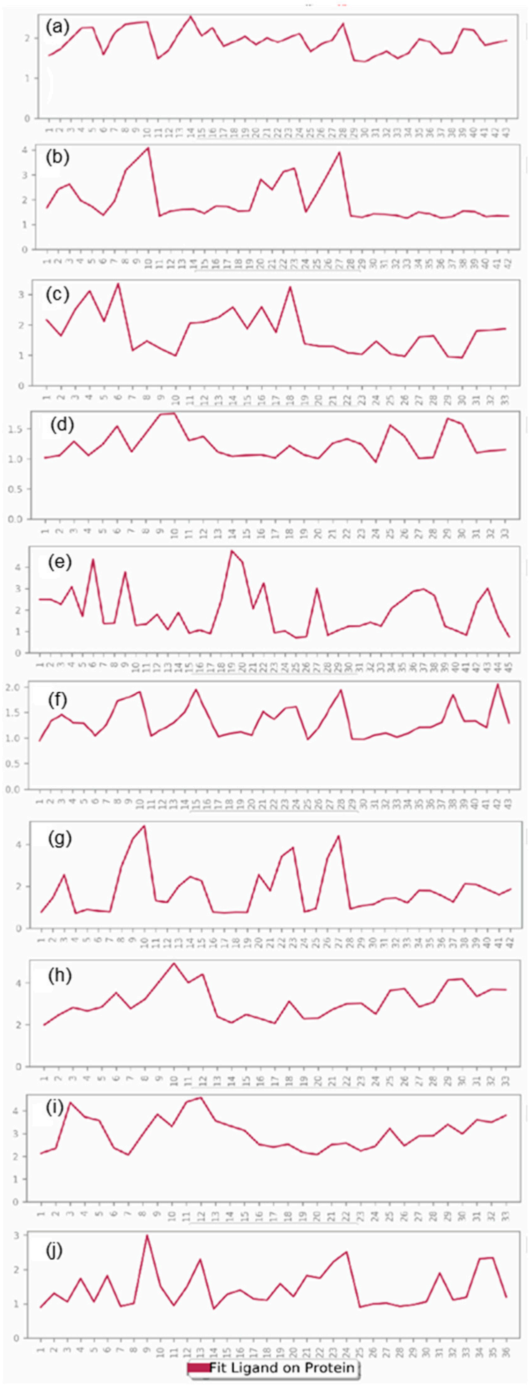

Figure S2. RMSF values plotted for the bioflavonoids in the docked complexes, i.e., (a) ZIKV^{Pro}-Rutin, (b) ZIKV^{Pro}-Nicotiflorin, (c) ZIKV^{Pro}-Isoquercitrin, (d) ZIKV^{Pro}-Hyperoside, (e) ZIKV^{Pro}-O7N (Control), (f) ZIKV^{RdRp}-Rutin, (g) ZIKV^{RdRp}-Nicotiflorin, (h) ZIKV^{RdRp}-Isoquercitrin, (i) ZIKV^{RdRp}-Hyperoside, (j) ZIKV^{RdRp}-Sofosbuvir (Control), fit with protein extracted from the respective 500 ns MD simulation interval.

S1.4.1. Protein-ligand interaction profiling

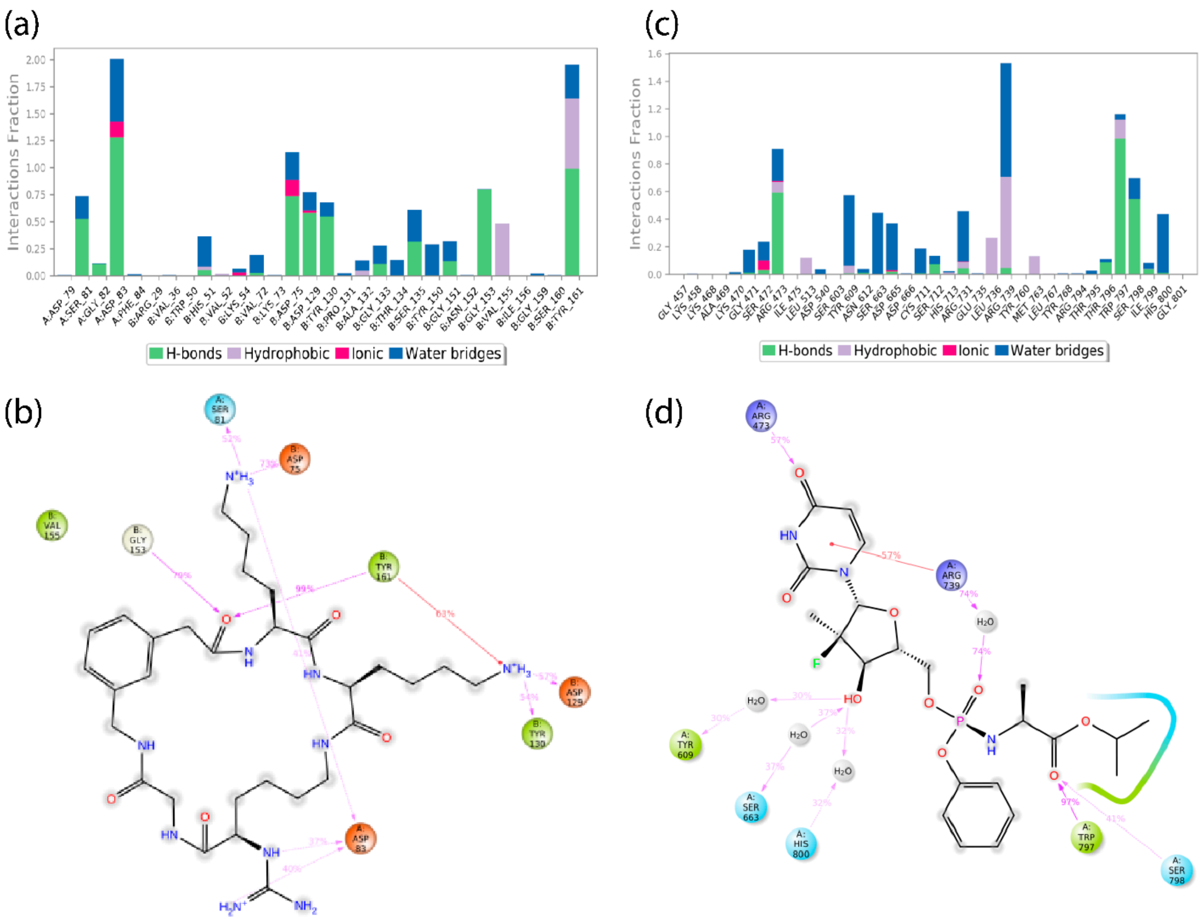

Figure S3. Protein-ligand interactions mapping for reference docked complexes, i.e., **(a-b)** ZIKV[Pro] – O7N, **(c-d)** ZIKV[RdRp]–Sofosbuvir inhibitor, extracted from 500 ns MD simulations. In 2D interaction diagram, the residues tyrosine, Valine, and Phenylalanine (green), Aspartic acid (red), histidine and asparagine (blue), and glycine (grey) exhibit the hydrophobic, negative, polar, and non-polar interactions, respectively along with hydrogen bonding (pink arrow) and pi-pi stacking (green line) with the receptor are extracted at 30% of the total MD simulation interaction interval.

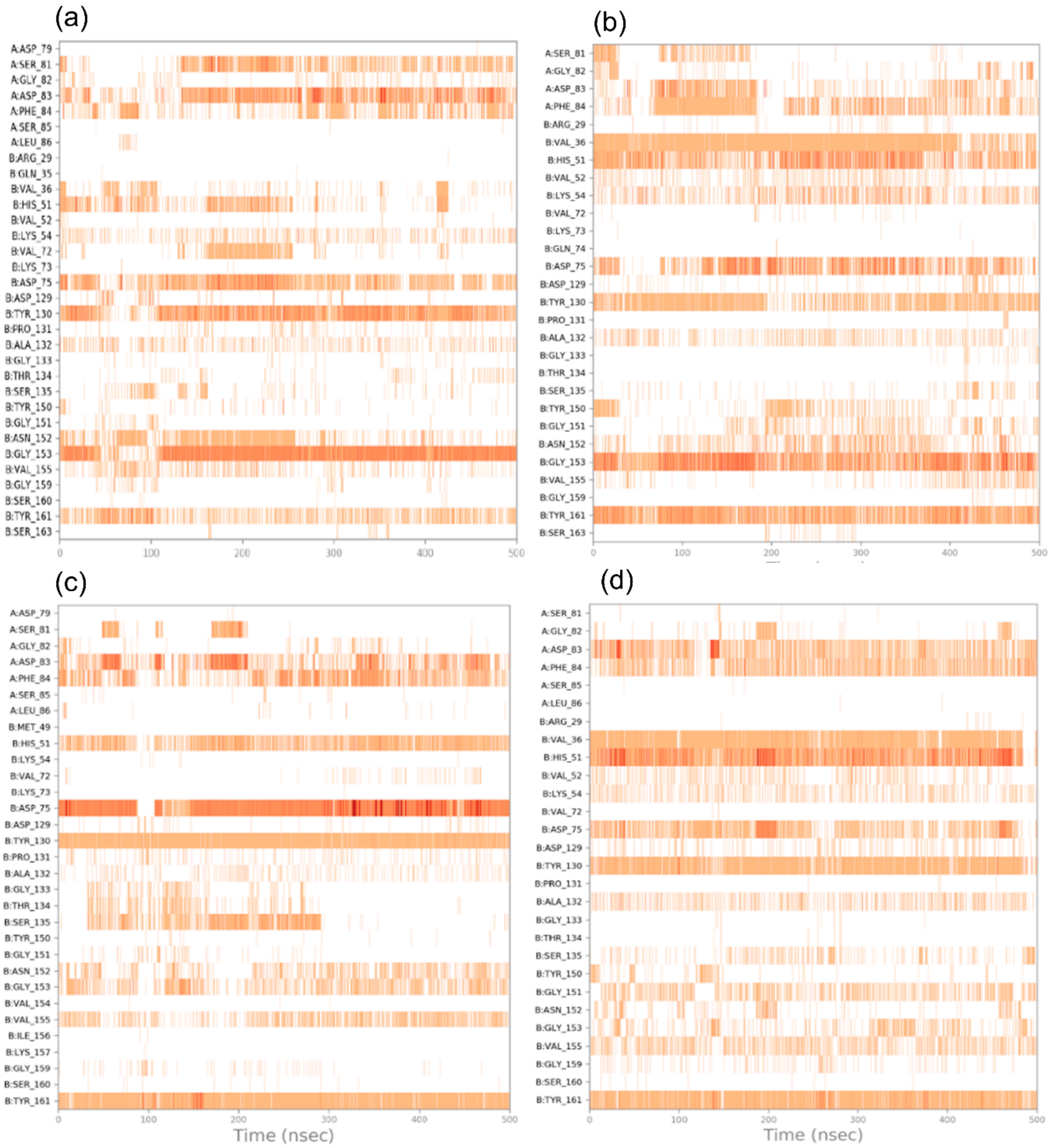

Figure S4. The panel shows which residues of ZIKV^{Pro} interaction with the selected ligands i.e., a. Rutin, b. Nicotiflorin, c. Isoquercitrin, and d. Hyperoside, in each trajectory frame, over the course of the 500 ns md trajectory. Some residues make more than one specific contact with the ligand, which is represented by a darker shade of orange, according to the scale to the right of the plot.

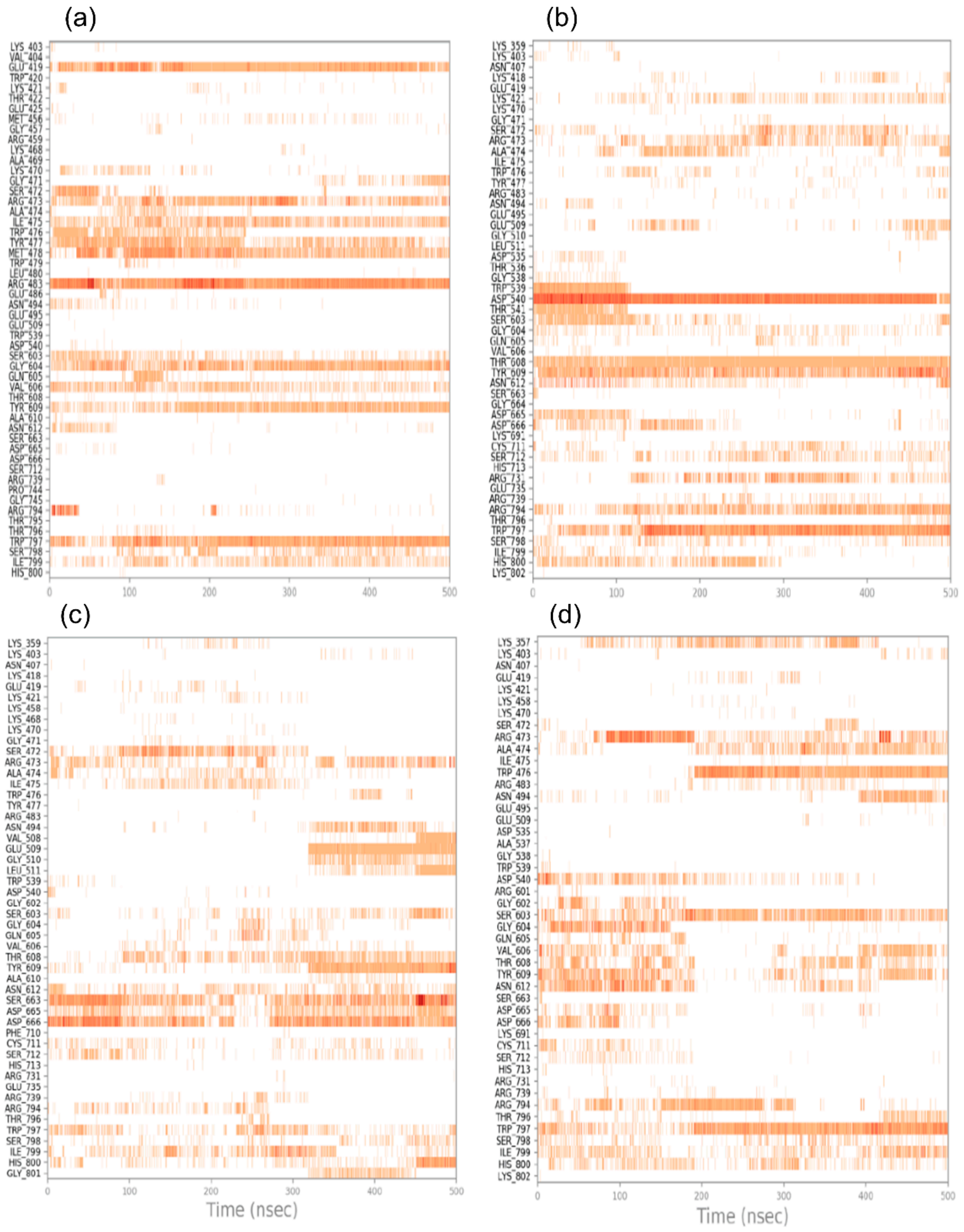

Figure S5. The panel shows which residues of ZIKV^{RdRp} interact with the selected ligands, i.e., **a.** Rutin, **b.** Nicotiflorin, **c.** Isoquercitrin, and **d.** Hyperoside, in each trajectory frame, over the course of the 500 ns md trajectory. Some residues make more than one specific contact with the ligand, which is represented by a darker shade of orange, according to the scale to the right of the plot.

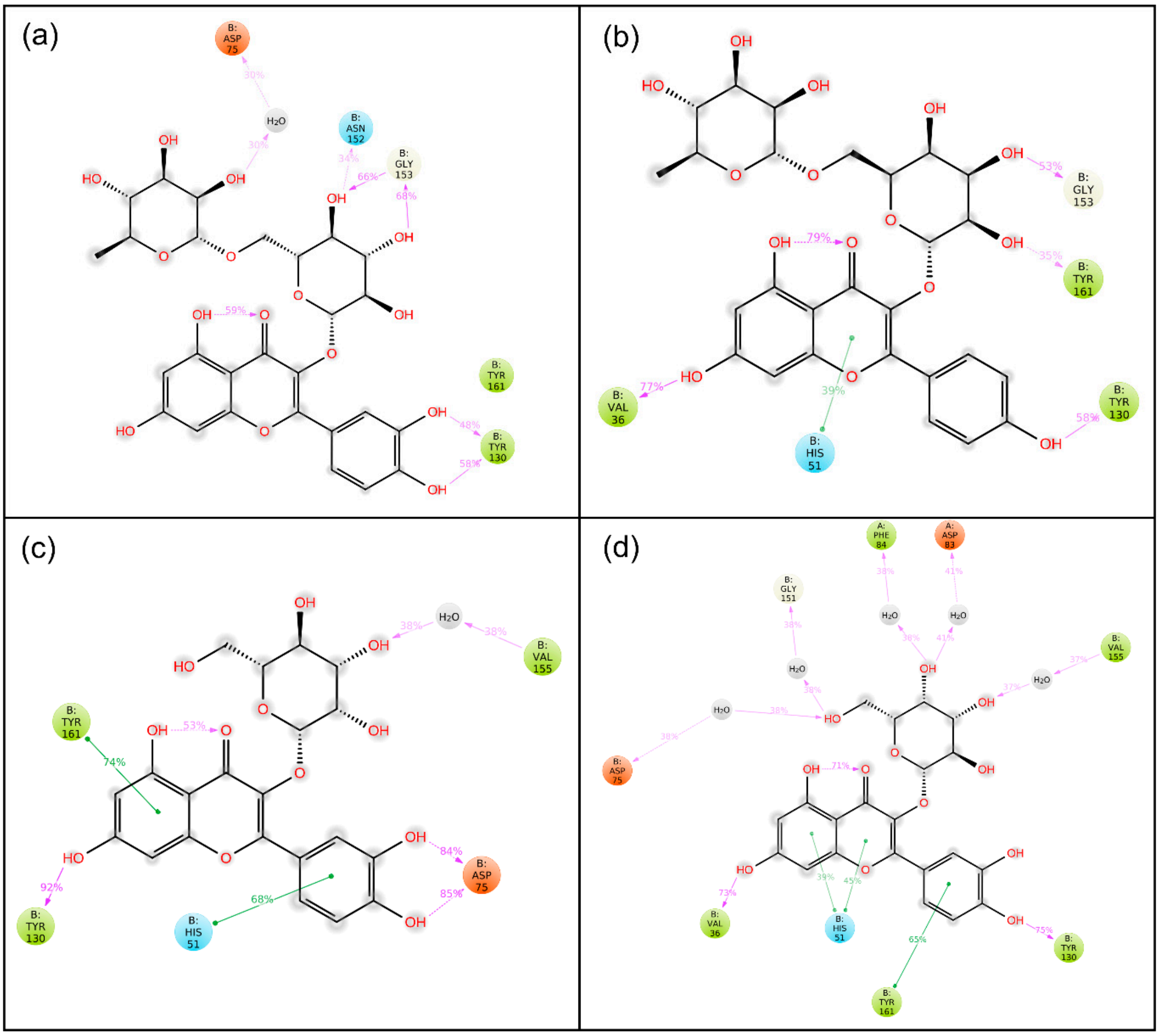

Figure S6. 2D interaction diagram of protein-ligand interactions maps for ZIKVPro with selected bioflavonoids, i.e., (a) Rutin, (b) Nicotiflorin, (c) Isoquercitrin, and (d) Hyperoside extracted from the total 500 ns MD simulations. Herein, residues tyrosine, Valine, and Phenylalanine (green), Aspartic acid (red), histidine and asparagine (blue), and glycine (grey) exhibit the hydrophobic, negative, polar, and non-polar interactions, respectively, along with hydrogen bonding (pink arrow) and π-π stacking (green line) with the receptor are extracted at 30% of the total MD simulation interaction interval.

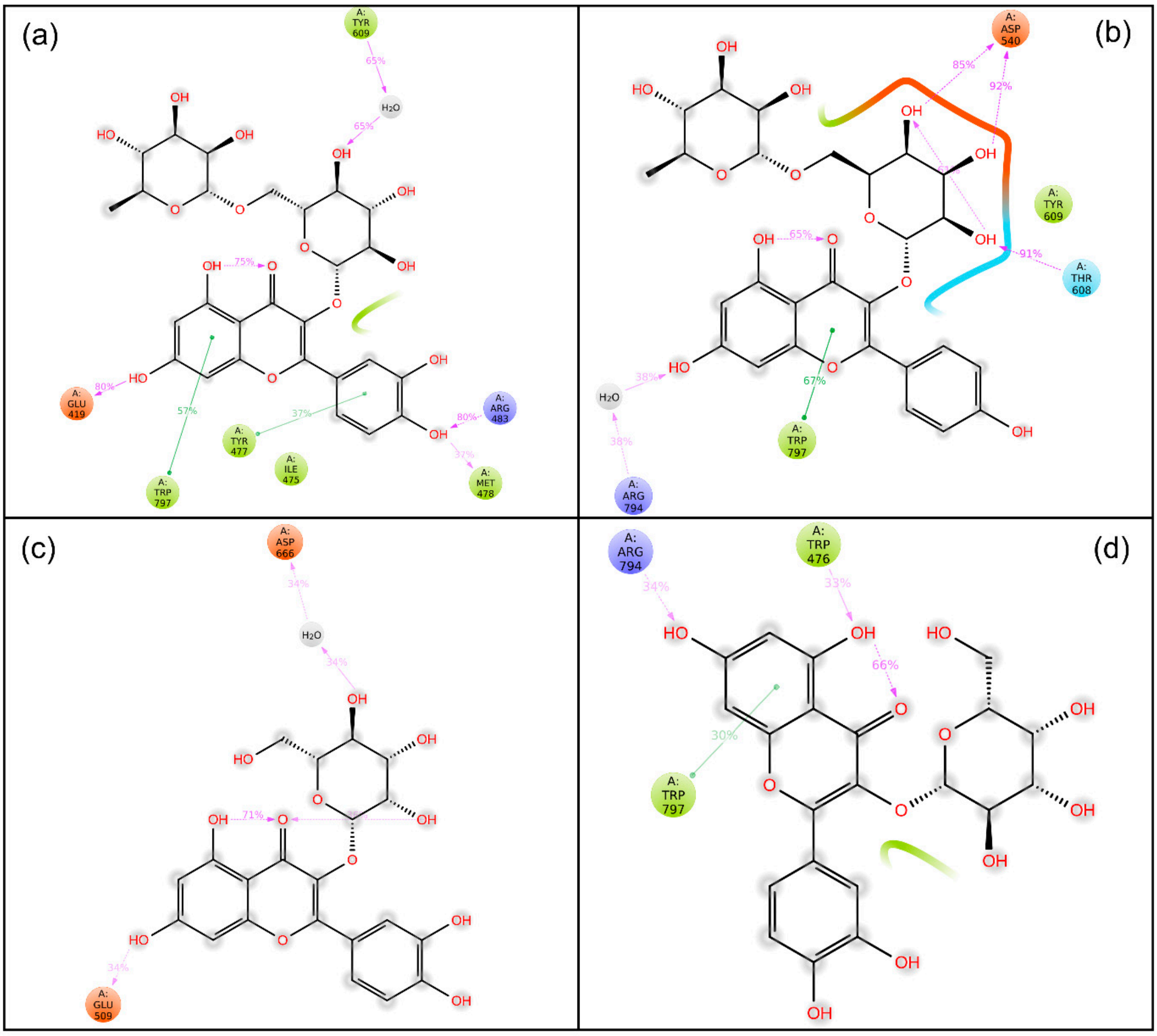

Figure S7. 2D interaction diagram of protein-ligand interactions mapping for ZIKV^{RdRp} with selected natural compounds, i.e. (a) Rutin, (b) Nicotiflorin, (c) Isoquercitrin, and (d) Hyperoside, extracted from 500 ns MD simulations. Residues tyrosine, Valine, and Phenylalanine (green), Aspartic acid (red), histidine and asparagine (blue), and glycine (grey) exhibit the hydrophobic, negative, polar, and non-polar interactions, respectively, along with hydrogen bonding (pink arrow) and pi-pi stacking (green line) with the receptor are extracted at 30% of the total MD simulation interaction interval.

S1.5 Endpoint free binding energy calculation

Table S8. Calculated energy components and net binding free energies (kcal/mol) values for ZIKV^{pro} and ZIKV^{RdRp} complex with selected bioflavonoids against reference compound snapshots collected from the respective 500 ns MD simulation trajectories.

MM/GBSA components	Energy (kcal/mol)									
	ZIKV^{pro}-Rutin	ZIKV^{RdRp}-Rutin	ZIKV^{pro}-Nicotiflorin	ZIKV^{RdRp}-Nicotiflorin	ZIKV^{pro}-Isoquercitrin	ZIKV^{RdRp}-Isoquercitrin	ZIKV^{pro}-Hyperoside	ZIKV^{RdRp}-Hyperoside	ZIKV^{pro}-O7N	ZIKV^{RdRp}-Sofosbuvir
ΔG_{Bind}	-57.09±6.61	-67.47±5.73	-39.42±8.45	-60.84±4.20	-48.32±4.58	-54.15±7.11	-44.53±6.46	-57.06±4.32	-74.37±6.44	-59.83±3.85
$\Delta G_{Bind\ Coulomb}$	-38.03±4.08	-31.87±8.21	-23.41±8.07	-19.78±6.04	-28.10±7.36	-24.38±4.91	-31.33±8.10	-19.98±8.23	-149.15±7.56	-28.19±4.43
$\Delta G_{Bind\ Covalent}$	1.95±2.46	5.45±3.16	4.97±2.91	6.04±1.36	3.34±1.94	2.28±0.95	3.73±1.09	2.15±1.13	2.72±2.10	3.24±0.96
$\Delta G_{Bind\ Hbond}$	-2.90±0.56	-2.37±0.52	-2.38±1.08	-2.09±0.66	-3.26±0.57	-2.54±0.33	-1.81±0.46	-1.37±0.52	-4.34±0.50	-1.91±0.62
$\Delta G_{Bind\ Lipo}$	-13.23±1.17	-17.14±1.18	-11.79±1.72	-17.70±1.84	-7.01±0.89	-11.37±1.35	-11.28±1.10	-12.46±0.97	-16.30±1.48	-15.01±0.78
$\Delta G_{Bind\ Packing}$	-1.42±0.48	-7.27±0.90	-2.82±0.85	-9.00±0.98	-5.12±0.44	-6.27±0.79	-2.38±1.21	-6.75±1.03	-0.18±0.29	-4.43±0.76
$\Delta G_{Bind\ Solv\ GB}$	38.83±3.335	45.49±4.06	29.87±4.41	39.76±1.90	27.30±6.34	37.67±3.01	31.67±5.35	29.48±5.92	139.16±6.46	35.60±3.36
$\Delta G_{Bind\ vdW}$	-42.29±4.49	-59.75±2.64	-33.85±5.58	-58.07±3.00	-35.47±2.23	-49.52±1.94	-33.12±5.17	-48.12±3.32	-46.28±3.45	-49.13±3.05
Ligand Strain Energy	7.40±2.47	5.66±3.37	9.25±4.05	6.15±2.72	7.99±3.32	4.14±1.82	4.07±1.74	2.60±2.07	7.80±2.92	9.92±2.41

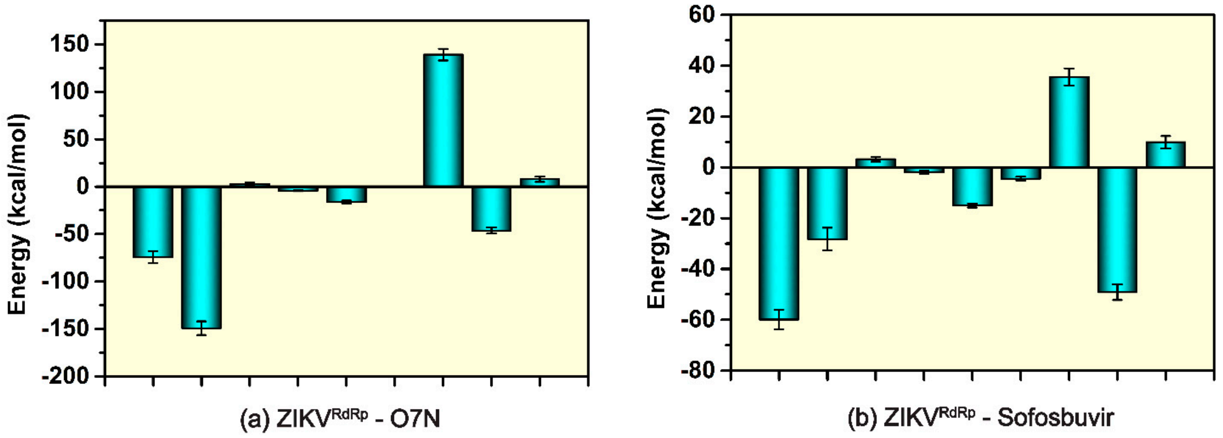

Figure S8. Calculated free energy components and net MM/GBSA binding free energy (kcal/mol) with standard deviation values for extracted snapshots of reference docked complexes, i.e., **(a).** ZIKV^{Pro}- O7N, **(b).** ZIKV^{RdRp} – Sofosbuvir, from respective 500 ns MD simulation trajectories.
